# Sea Turtle Nesting Beach Fidelity Revealed by Satellite Tracking

**DOI:** 10.1002/ece3.74341

**Published:** 2026-10-06

**Authors:** Nupur Kale, Graeme C. Hays, Jeanne A. Mortimer, Anna Zora, Kimberley L. Stokes, Nicole Esteban

**Affiliations:** ^1^ Department of Biosciences Swansea University Wales UK; ^2^ Deakin Marine Research and Innovation Centre, School of Life and Environmental Sciences Deakin University Geelong Victoria Australia; ^3^ Independent Researcher Victoria, Mahé Republic of Seychelles; ^4^ Department of Biology University of Florida Gainesville Florida USA; ^5^ Frégate Island Foundation, Angelfish Marina Roche Caiman, Mahé Republic of Seychelles

**Keywords:** behaviour, endangered species, Fastloc‐GPS, inter‐nesting, reproduction

## Abstract

Site fidelity describes the tendency to return to or reuse familiar sites, yet some animals may use alternative sites while maintaining fidelity to primary locations, with consequences for population estimates as well as resilience to changing environments. Here we assessed and compared nesting beach fidelity of hawksbill turtles ( *Eretmochelys imbricata* ) on Frégate Island, Seychelles using data from beach foot patrols (2017–2025; *n* = 575 turtles) and satellite telemetry (2023; *n* = 10 turtles) and then examined the implications of fidelity strategies for population estimates. We also reviewed published literature on nesting beach use across sea turtle species. Satellite tracking showed that 50% of equipped turtles were study‐beach faithful, laying clutches on one beach, and 50% were study‐beach unfaithful, nesting across 2–4 beaches on Frégate and on nearby islands. Consequently, clutch frequency estimated through tracking was 1.9 times higher than foot patrol estimates. Estimates of adult female abundance increased as the assumed proportion of clutches laid on other beaches increased, independent of clutch frequency. Across 51 studies at 43 sites around the world, study‐beach unfaithful individuals were more likely to be recorded nesting at mainland beaches (21.4% compared with 9.3% on island beaches), with mainland surveys covering greater distances (1.6–1000 km) than island surveys (1–15.9 km). Maximum inter‐nest distances varied across species: green turtles ( *Chelonia mydas* ; 2250 km), leatherbacks ( *Dermochelys coriacea* ; 1849 km), loggerheads ( *Caretta caretta* ; 800 km), olive ridleys ( *Lepidochelys olivacea* ; 320 km), and hawksbills (300 km). We demonstrate mixed fidelity strategies within an island hawksbill population, suggesting that fidelity reflects natal philopatry and the proximity of alternative sites. Limited monitoring coverage at remote sites likely contributes to under‐detection of nestings by study‐beach unfaithful individuals. Individuals using alternative sites signal behavioural plasticity that may contribute to resilience against habitat loss. We recommend characterising beach fidelity through regional data sharing and satellite tracking to inform reliable demographic inference and identify subpopulations vulnerable to climate change.

## Graphical abstract

Our study demonstrates mixed fidelity strategies within an island hawksbill population, suggesting that fidelity reflects natal philopatry and the proximity of alternative sites. Limited monitoring coverage at remote sites likely contributes to under‐detection of nestings by study‐beach unfaithful individuals. Individuals using alternative sites signal behavioural plasticity that may contribute to resilience against habitat loss. We recommend characterising beach fidelity through regional data sharing and satellite tracking to inform demographic inference and identify subpopulations vulnerable to climate change.

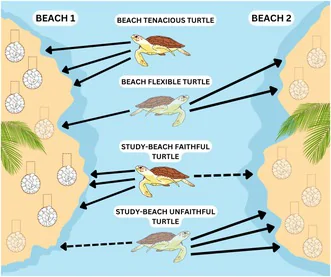

## 1 Introduction

Site fidelity—the behavioural tendency to return to familiar sites—is common across taxonomic groups (Switzer 1993) including mammals (e.g., Black‐tailed deer; Kreling et al. 2021), reptiles (e.g., Burmese pythons; Smith et al. 2021) and birds (e.g., Lesser kestrels; Ramellini et al. 2022). This behaviour emerges through multiple mechanisms including genetic imprinting and spatial memory, often resulting in strong spatial affinity (Berthold et al. 1992; Fagan et al. 2013). In stable environments, maintaining fidelity can provide benefits such as predictable foraging resources and reduced predation risk (Shimada et al. 2020; Gehr et al. 2020). Recent research suggests that strong fidelity to a single site may represent an ‘ecological trap’ preventing animals from exploring and occupying optimal habitats, even reducing survival rates (Merkle et al. 2022). Some individuals are known to adjust their fidelity to include multiple sites or adopt a ‘win‐stay, lose‐switch’ strategy, changing habitats during unpredictable environmental conditions while revisiting the same site if outcomes remain positive (Lafontaine et al. 2017). While these strategies offer behavioural plasticity, changing sites can incur energetic and survival costs, including increased metabolic expenditure and potential exposure to unknown predation risks or environmental challenges (Schmidt 2014; Gerber et al. 2019). Nevertheless, understanding whether a population possesses the flexibility to use alternative sites may provide insights into its capacity to persist under climate change‐driven habitat loss.

Female sea turtles are generally believed to show strong fidelity to their nesting sites (Carr 1975) returning (or remigrating) to nest on the same beach across different nesting seasons. However, turtles may sometimes use more than one nesting beach; for example, individuals from a single loggerhead ( *Caretta caretta* ) subpopulation nested on beaches 290 km apart in Florida, USA, and 377 km apart in Japan within a season (Bjorndal et al. 1983; Iwamoto et al. 1985). The generally accepted view of the life history of nesting sea turtles was challenged over 30 years ago with a different conceptual model suggesting that turtles encountered on any particular nesting beach consist of (i) individuals that routinely use that beach both within and across seasons, and (ii) individuals that have strayed from another beach elsewhere and so are only seen rarely within and across seasons because they routinely nest elsewhere (Hays and Sutherland 1991). Here we define these two types of turtles that can be seen on an individual beach as ‘study‐beach faithful’ and ‘study‐beach unfaithful’. Similarly, Richardson (1982) described these two sets of individuals as turtles seen once and having a low chance of being resighted in a subsequent year, and turtles seen several times in a season and having a high chance of returning. Supporting this conceptual model, Hays and Sutherland (1991) used flipper‐tagging records to show that turtles seen nesting multiple times in a single season (study‐beach faithful turtles) on a beach in Greece had an 80% chance of being observed as a remigrant (i.e., seen nesting in a subsequent year), whereas turtles seen nesting only once in a season (study‐beach unfaithful turtles) had a 13% chance of being seen as a remigrant. Early satellite tracking studies also provided support for this model (Hays et al. 1991). However, establishing the generality of these findings has been constrained by traditional methods, notably foot patrols and flipper tag returns that are labour‐ and time‐intensive, often limiting surveys to a few beaches and obscuring understanding of turtle spatial use across a broader scale. Hence our understanding of nesting beach fidelity in sea turtles across species and the consequences of fidelity for demographic estimation remains limited.

Beyond the methodological limitations of observing animal movements, another challenge in assessing fidelity lies in defining what constitutes beach fidelity. On islands, nesting beaches are either continuous expanses of sand with no clear boundaries, or discrete patches with varying degrees of spatial isolation separated by physical barriers such as cliffs or rock patches. Depending on the geographic context, fidelity can be measured using distinct models. For remote and isolated islands separated from any landmass by 1000s of km, previous studies have assessed levels of fidelity in fine‐scale movements between beaches on the same island (e.g., beach clusters on Ascension Island; Mortimer and Portier 1989). For islands that are separated from each other by < 1 km to 100s of km, for example, in archipelagos, fidelity can occur at two levels: (1) ‘island fidelity’ where turtles utilise beaches scattered around the same island, and (2) ‘beach fidelity’ where turtles use the same individual beach across nesting events. The factors that influence the extent to which fidelity operates across multiple spatial scales remain underexplored for island systems.

The use of satellite telemetry can overcome methodological and geographical constraints of traditional patrol methods, enabling a more comprehensive characterisation of nesting beach fidelity (Tucker 2010). Here, we examine nesting beach fidelity in the critically endangered hawksbill turtle ( *Eretmochelys imbricata* ) in the Republic of Seychelles. We estimate life history parameters (clutch frequency, inter‐nesting interval) using data from foot patrols and compare them to estimates from Fastloc‐GPS tracking data, in order to explore the implications of a mismatch in assumptions of beach fidelity for inference of adult female abundance. We then compile peer‐reviewed literature on study‐beach unfaithful sea turtles (i.e., where nesting occurred on more than one beach) to understand fidelity patterns in sea turtles. Finally, we discuss the implications of fidelity strategies for population resilience under climate change.

## 2 Materials and Methods

### 2.1 Foot Patrols to Record Nesting Activities

Nesting data were collected by patrolling sandy pocket beaches separated by rocky headlands on Frégate Island (4.58° S, 55.94° E), Republic of Seychelles (Figure 1) each day during the nesting season from 1 October to 20 March (2017–2018 to 2024–2025). Foot patrols were conducted between 08:00 and 18:00 h as hawksbill turtles predominantly nest during the day in the Seychelles (Mortimer and Bresson 1999). After encountering a turtle, observers waited until the turtle completed oviposition before measuring the carapace to the nearest 0.1 cm (curved carapace length notch to tip, CCL_n‐t_, hereafter called CCL, from the anterior edge of the middle of the nuchal scute where it meets the skin of the neck to the posterior tip of the longest supracaudal scute; Bolten 1999). At first encounter, the turtle was tagged on each front flipper using Titanium turtle tags (Stockbrands, Australia). Recorded data included the time and date of encounter, confirmation of oviposition, beach name, and tag numbers on each flipper.

**FIGURE 1:**
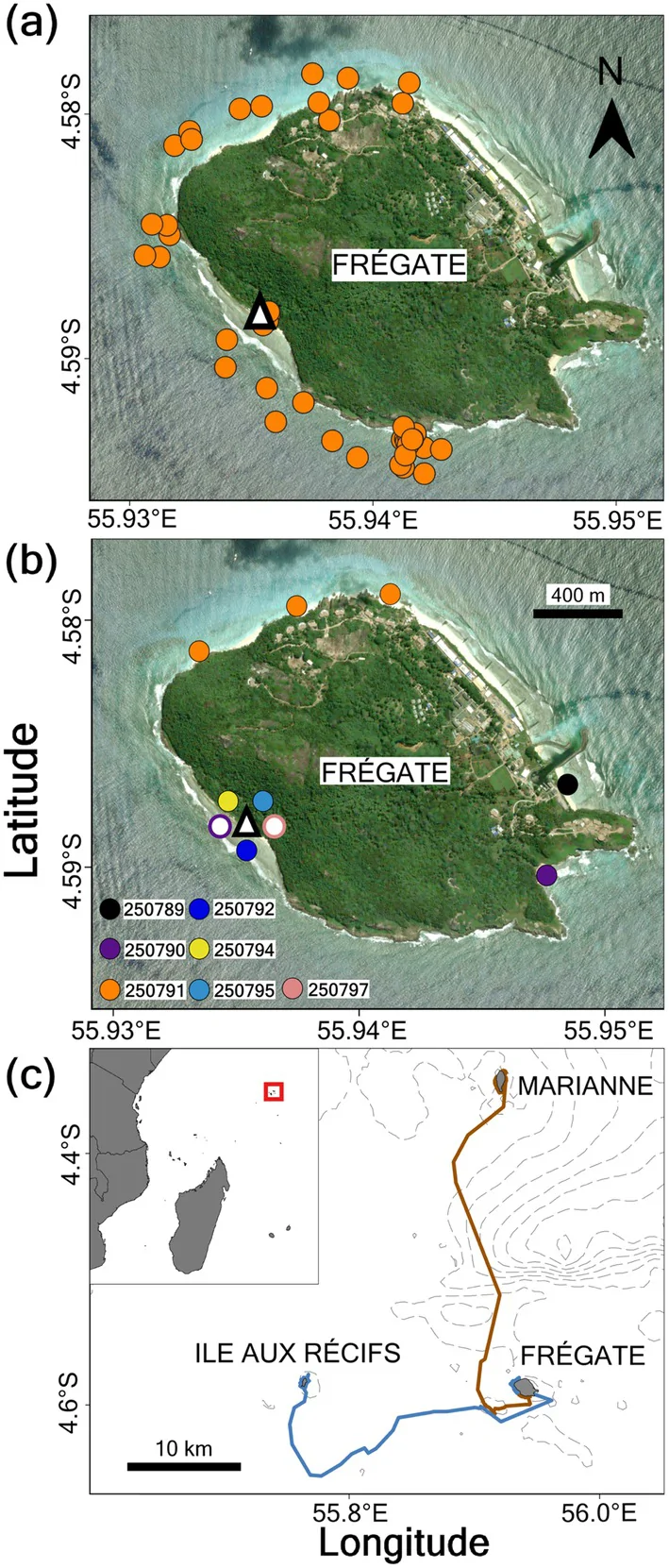
Hawksbill turtle ( *Eretmochelys imbricata* ) nesting beach fidelity was recorded and assessed using satellite telemetry at Frégate Island, Republic of Seychelles. (a) Example of Fastloc‐GPS locations of turtle 250791 during the inter‐nesting period: The turtle was tagged on Grand Anse beach (open triangle), and subsequent nesting events (orange circles on panel b) were deduced from haul‐out data (e.g., periods when an individual is on land to nest) and Fastloc‐GPS location data. (b) Ten satellite‐tagged individuals nested on Grand Anse beach before tag attachment (open triangle); four turtles were study‐beach faithful, repeatedly nesting on Grand Anse (open triangle), five individuals were study‐beach unfaithful, changing sites to nest on Marina beach (black circle), Anse Parc (purple circle) and Anse Victorin, Anse Macquereau and Anse Bambous (orange circles) or on other islands (panel c), and one turtle did not renest. Filled and open circles indicate one and two clutches respectively. (c) Turtles 250793 and 250796 (brown and turquoise tracks respectively) relocated to lay four and three clutches on Marianne and Ile aux Récifs Islands within the inner granitic islands of Seychelles. Bathymetry contours (dashed lines) at 25 m intervals were extracted from GEBCO 15 arc‐second grid (GEBCO 2026). Inset shows the location of the inner islands of Seychelles (red box) in the Western Indian Ocean.

### 2.2 Satellite Tracking During the Nesting Season

Ten adult hawksbill turtles were equipped with Fastloc‐GPS Argos satellite tags (SPLASH10‐BF, Wildlife Computers, Seattle, USA) at Grand Anse beach, Frégate Island during the peak nesting period between 30 November and 8 December 2023. After oviposition, CCL was measured and each turtle was prevented from returning to the sea using an open‐top wooden box for tag attachment. The carapace was lightly sanded and cleaned with isopropyl ethanol, and tags were attached using epoxy (Pure 150‐PRO, Dewalt, Germany) and coated with anti‐fouling paint (Trilux 33, International, Netherlands). The process took approximately 2 h, after which the turtle returned to the sea (Esteban et al. 2017).

Satellite tags were programmed to detect and register haul‐out events, (which include periods when an individual is on land nesting or attempting to nest; Esteban et al. 2017) after an emergence duration of 20 min. The location, date and time of each haul‐out was then included in tag transmission messages to the Argos system. Given that the minimum nesting emergence is typically 45 min on Frégate Island, setting 20 min to define a haul‐out ensured that all nesting emergences would be registered by the tags. Visual examination of the plotted tracks was used to identify the point of departure to their foraging grounds. This point was indicated by turtles travelling in a persistent direction over a prolonged period away from their final nesting site. All location data after this time were excluded from analyses. Fastloc‐GPS locations were filtered to include those obtained with a minimum of 5 satellites and a residual value < 35, providing location accuracy within tens of metres (Dujon et al. 2014). Nesting events were identified using: (i) haul‐out data, (ii) Fastloc‐GPS locations on land, and (iii) days elapsed between haul‐outs. Further, we checked location points immediately before and after each haul‐out event, verifying that timestamps fell within 25–30 min of the haul‐out and that the nearest beach was within plausible distance, to confirm that the turtle spent time on land. Isolated haul‐outs separated by intervals consistent with the range of inter‐nesting interval for the region (7–23 days; Mortimer and Bresson 1999) were treated as nesting events. In case of multiple haul‐outs within a 1–3‐day period, the final haul‐out was categorised as the nesting event.

The nesting event classification was validated using direct observations and/or comparison to the published mean inter‐nesting interval for the Seychelles (15.1 days; Mortimer and Bresson 1999). Where available, flipper tag numbers of satellite‐tagged individuals were checked against foot patrol records to collate their nesting history on Frégate Island.

### 2.3 Estimation of Clutch Frequency

We compiled annual clutch counts for each turtle that nested on Frégate Island using flipper‐tag records from 2017 to 2025. Using foot patrol data, we calculated mean observed clutch frequency (OCF) per nesting season as the average number of clutches recorded for each turtle between 2017 and 2025. Prior to calculating estimated clutch frequency (ECF), we determined the typical range of inter‐nesting intervals in the region as 12–17 days (Mortimer and Bresson 1999). Where intervals between two observed clutches exceeded 24 days (i.e., twice the minimum interval of 12 days), we assumed that individuals laid a ‘missed’ clutch during that period. We estimated these missed clutches by dividing the observed interval by the previously published mean inter‐nesting interval (15.1 days; Mortimer and Bresson 1999) and then added the obtained nearest integer value to the first observed clutch to calculate ECF (Johnson and Ehrhart 1996).

For satellite‐tagged turtles, we first calculated the length of residency at the breeding ground for each turtle that is, days elapsed between the first observed nesting event in 2023–2024 (determined through foot patrols for individuals that nested earlier in the season) and the departure date from breeding grounds from tracking data (Table S1). We then calculated the ECF by dividing the residency period by the mean inter‐nesting interval of satellite‐tracked turtles in our study and adding one clutch to account for the first nesting event (following methods described in Esteban et al. 2017).

All statistical analyses were performed in R version 4.2.3 (R Core Team 2026). Data were tested for normality using the Shapiro–Wilk test. Values are reported as mean ± SD, range. We compared ECF between nesting seasons and satellite‐tagged turtles using the non‐parametric Kruskal‐Wallis test and post hoc Dunn's test adjusted with the Bonferroni method.

### 2.4 Quantifying Beach Fidelity

We used nest locations identified from satellite telemetry to quantify the extent of beach fidelity of satellite‐tagged turtles to the tag deployment site, calculated as the straight‐line distance between the clutch laid on the day of tag attachment at Grand Anse and each subsequent nest location. For nests with uncertain locations on Grand Anse (*n =* 3) due to the absence of associated Fastloc‐GPS locations when a haul‐out was recorded, we assigned a distance of 0 km from the first nest location. We calculated the incidence of breakdown in fidelity as the percentage of clutches laid on beaches other than Grand Anse, which is a 300‐m long beach bordered by rocky headland.

Using nest locations from foot patrols, we calculated the proportion of turtles showing beach fidelity across subsequent seasons based on whether individuals laid 1 or > 1 clutch on Grand Anse. To allow sufficient time to elapse for remigration, only turtles that nested in 2017–2018 and 2018–2019 were included in this analysis (remigration interval in Seychelles = 2–3 years, Mortimer and Bresson 1999). We used a G‐test to compare the observed and expected proportion of remigrant turtles that laid 1 versus > 1 clutch.

### 2.5 Implications of Beach Fidelity for Adult Female Abundance

We assessed how estimates of the number of adult female turtles using Grand Anse varied with the assumed proportion of clutches laid on other beaches, under different ECF derived using results from foot patrols and satellite tracking. We used the equation describing the relationship between clutch counts and estimated total adult females from Ceriani et al. (2019):

$${\mathrm{TF}}_{y}={\left(n_{y}/c\right)}^{*}\;r$$

where TF_y_ is the estimated total number of adult females in year y, *n*_y_ is the clutch count in year y, *c* is clutch frequency and *r* is remigration interval.

We modified *n*_y_ to account for differences in beach fidelity since the clutch counts recorded at Grand Anse (*n*_obs_) were a proportion of clutches laid (*f*) relative to all other beaches.

$$n_{y}=n_{\mathrm{obs}}/f$$

Hence, TF_*y*_ = (*n*_obs_/(*c* * *f*)) * *r*.

For the number of clutches observed (*n*_obs_), we used the number of clutches recorded during foot patrols on Grand Anse in 2023–2024. For remigration interval, we used the published estimate of 2 to 3 years for hawksbills in the region (Mortimer and Bresson 1999). We defined the parameter *e* as the proportion of clutches laid elsewhere, that is, *e* = 1−*f*, using *e* values between 0 and 0.9. We then estimated adult female abundance across the range of *e* for three ECF: (1) derived from foot patrol data, (2) the maximum annual value from foot patrol data, and (3) derived from satellite tracking data.

### 2.6 Literature Review

We searched for studies reporting study‐beach unfaithful individuals recorded within or between seasons for all sea turtle species using the Web of Science in June 2026 (1950–2026). The keywords used to shortlist studies were: (‘sea turtles’ AND (‘inter‐nesting’ OR ‘nesting’) AND (‘site fidelity’ OR ‘beach variance’)). We used Google Scholar for forward and backward searches of citations of the shortlisted studies, scanning for information on fidelity. We extracted data on species, nesting geography, oceanic region, methods, sample sizes, number of individuals that used multiple beaches, clutches laid within or between seasons, primary nesting site and country, total distance covered by monitoring efforts, secondary site/s and the reported maximum distance between clutches.

## 3 Results

### 3.1 Clutch Frequency

Mean OCF calculated from foot patrols between 2017–2018 and 2024–2025 was 1.6 clutches (±0.2, 1–6 clutches, *n* = 575 individuals). The mean ECF across the study period was 1.9 clutches (±0.3, 1–6 clutches, *n* = 575 individuals). In comparison, mean ECF from satellite telemetry was 3.7 clutches (±1.3, 1.2–4.9 clutches) with > 1 clutch laid by 9 individuals (of *n* = 10 individuals; Figure 2 and Table S2).

**FIGURE 2:**
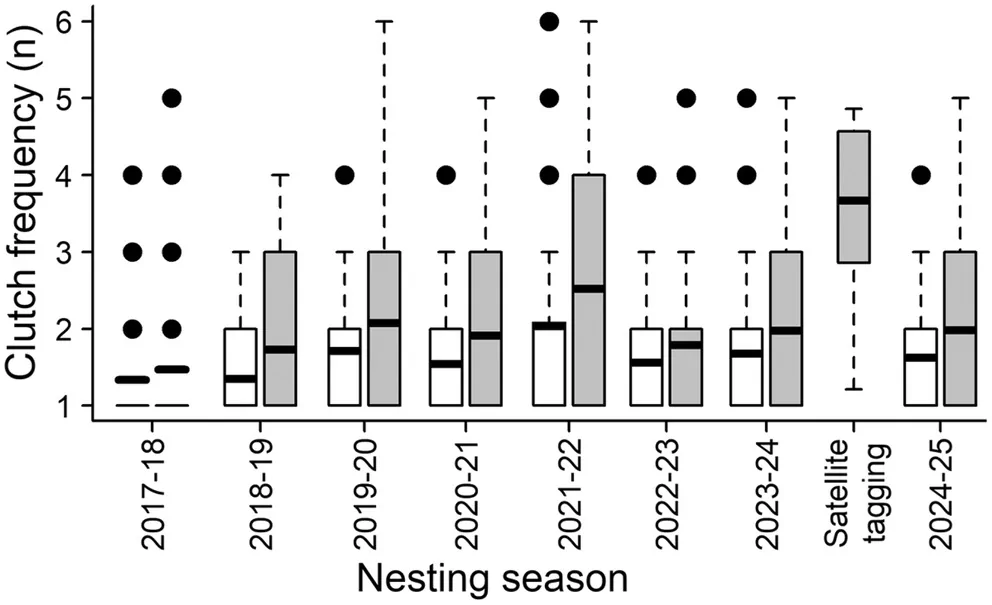
Mean observed and estimated clutch frequencies (OCF and ECF; white and grey boxes respectively) of hawksbill turtles recorded by patrolling beaches on Frégate Island, Seychelles were 1.6 and 2 clutches respectively, almost 50% lower than the 3.7 clutches estimated using satellite tracking data in 2023–2024. Mean clutch frequencies across nesting seasons are indicated as bold horizontal black lines, boxes delineate the upper and lower quartiles, and whiskers show the data range. Outliers are represented by separate points.

ECF from satellite telemetry was higher than foot patrol ECF across nesting seasons (*H*_8_ = 33.2, *p* < 0.05; except 2021–2022, *p =* 0.61). Post hoc comparisons showed differences in ECF from foot patrols between 2017–2018 and 2021–2022 seasons (*p* < 0.05).

### 3.2 Extent of Beach Use

The movements of satellite‐tagged turtles were analysed using 1552 filtered Fastloc‐GPS locations (*n =* 171 locations excluded), tracked over 3–53 days (27 ± 17 days) before departing from the breeding grounds (Table S1). Seven turtles renested on Grand Anse or other beaches on Frégate Island (Figure 1b) while one turtle migrated to its foraging grounds directly after tag attachment. Turtle 250793 travelled 27.3 km north to Marianne Island and Turtle 250796 settled at Ile aux Récifs after travelling a straight‐line distance of 19 km to lay subsequent clutches (Figure 1c and Table S3).

Based on haul‐outs and Fastloc‐GPS positions, the mean inter‐nesting interval was 13.8 days (±1.3, 12–17 days, *n* = 19). Foot patrols validated two of the renesting events recorded on Frégate Island (Table S3).

### 3.3 Beach Fidelity

Of the subsequent 19 clutches laid by satellite‐tagged turtles, 37% were within 0.3 km of the first clutch (i.e., approximately the length of Grand Anse; Figure 3a and Table S3).

Flipper‐tagged individuals that laid > 1 clutch in 2017–18 and 2018–19 were more likely to remigrate to Frégate Island in subsequent seasons than individuals that were recorded laying only one clutch (*G*_1_ = 5.21, *p* = 0.02, *n* = 45 individuals; Figure 3b and Table S4). 91% of turtles that laid > 1 clutch on Grand Anse in 2017–2018 and 2018–2019 remigrated compared to 56% of turtles that were observed laying only 1 clutch. For example, satellite‐tagged turtles 250790 and 250797 had each been recorded laying four clutches per season on Grand Anse in previous seasons and up to four clutches on Grand Anse whilst being satellite tracked in 2023–2024 (Table S3). Turtles 250789 and 250796 had previously laid a single clutch on Grand Anse within a season, and each laid only one clutch on Grand Anse in 2023–2024. A third pattern was demonstrated by turtle 250791 which was recorded using multiple beaches on Frégate Island across seasons; she laid one clutch each at two beaches in 2017–2018 and one clutch each at four different beaches in 2023–2024 (Table S3).

**FIGURE 3:**
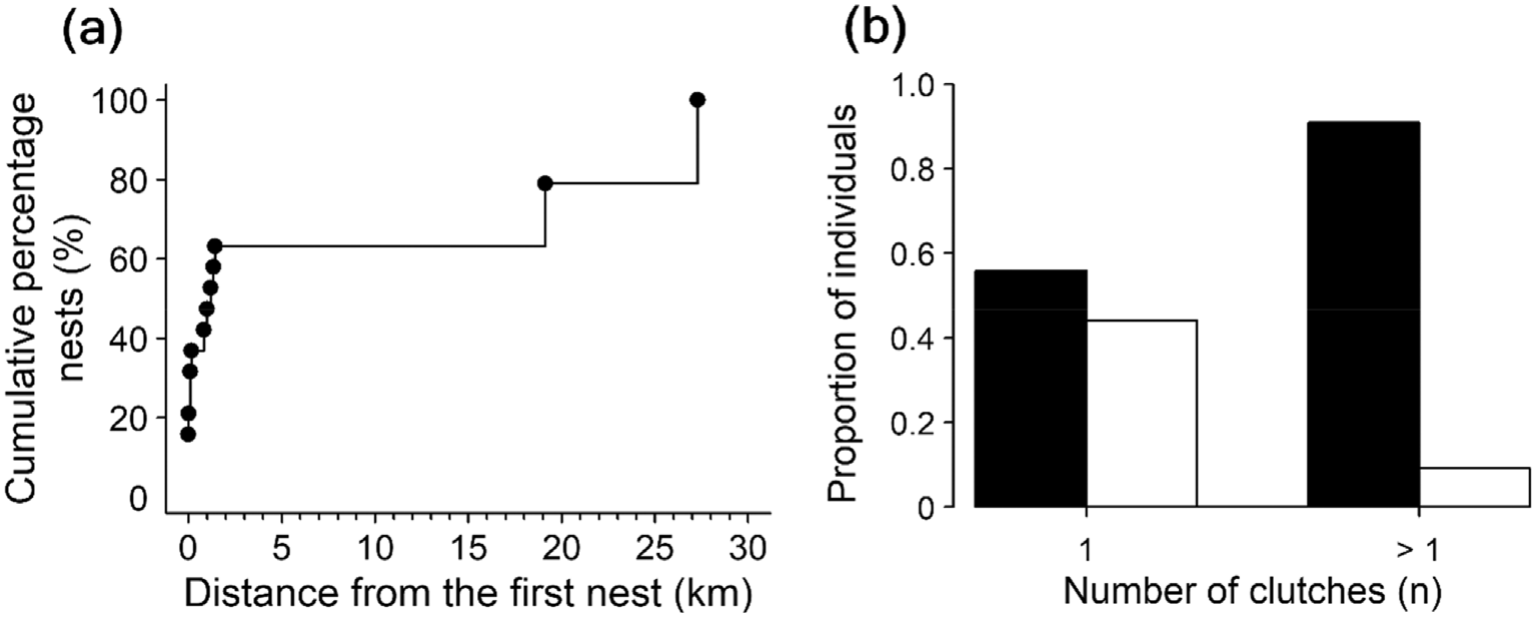
(a) Straight‐line distance between the first clutch and subsequent clutches indicated that 37% of clutches (*n* = 7 out of 19) from satellite‐tagged hawksbill turtles (*n* = 10) on Frégate Island were laid at Grand Anse beach, that is, within 300 m. (b) The proportion of flipper‐tagged individuals that remigrated (black bars) to Grand Anse and individuals that were not recorded in subsequent years (white bars) were compared between those that laid 1 and > 1 clutch. 91% of turtles that laid > 1 clutch on Grand Anse in 2017–2018 and 2018–2019 remigrated compared to 56% of turtles that were observed laying only 1 clutch.

### 3.4 Consequences of Mixed Fidelity on Abundance Estimates

As fidelity to the nesting beach of Grand Anse decreased, there was a corresponding increase in the estimated number of adult females using Grand Anse (Figure 4). Assuming that 60% of clutches were laid elsewhere based on our telemetry data, estimated adult female abundance was 2.5‐fold higher than when assuming all clutches are laid at Grand Anse (Table S5). This pattern was consistent across all clutch frequency scenarios, and abundance estimates were higher when using foot patrol‐derived ECF compared to those derived from telemetry‐based ECF (Figure 4). For example, 198 clutches were observed on Grand Anse in 2023–2024. If turtles laid their clutches exclusively on Grand Anse, then the foot patrol‐derived clutch frequency of 1.9 clutches per season and an average remigration interval of 2.5 years yield the estimated number of adult females using that beach as (198/1.9)*2.5 = 260 individuals. In contrast, if turtles using Grand Anse laid 60% of their clutches on other beaches, then the estimated number of adult females using that beach, at least sometimes, increases to (198/(1.9*0.4))*2.5 = 651 individuals. Similarly, using a clutch frequency of 3.7 clutches per season and assuming all clutches are laid on Grand Anse yields an estimate of 134 individuals, which rises to 334 individuals when 60% of the clutches are laid elsewhere.

**FIGURE 4:**
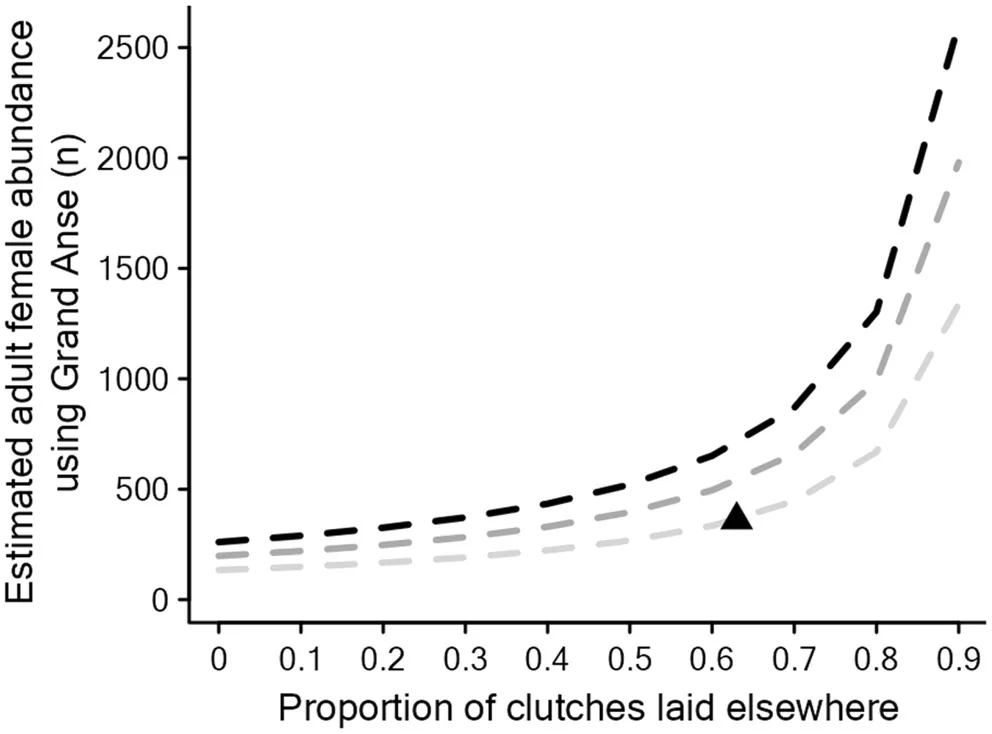
The predicted number of adult females using Grand Anse as a function of nesting beach fidelity indicates that abundance estimates increase when fidelity (defined as the proportion of clutches laid outside the study beach) is accounted for, regardless of estimated clutch frequency method. Dashed lines represent abundance estimates based on mean estimated clutch frequency (ECF) derived from foot patrol data (1.9 clutches; black), the maximum annual ECF from foot patrol data (2.5 clutches; dark grey), and satellite telemetry ECF (3.7 clutches; light grey). Black triangle indicates the estimated adult female abundance using Grand Anse (361 individuals), based on satellite tracking data showing that 63% of clutches were laid on other beaches.

### 3.5 Conceptual Framework for Observed Mixed Nesting Beach Fidelity

We conceptualised sea turtle beach use revealed by satellite tracking and flipper tagging as showing two behavioural strategies: beach tenacious individuals exhibit strong site fidelity by nesting exclusively on a single beach (adapted from site tenacity; Weishampel et al. 2003), while beach flexible individuals may use multiple beaches within and between seasons. Within a study area, turtles may appear as either study‐beach faithful or study‐beach unfaithful depending on the size of the monitored site relative to the extent of turtle fidelity. Study‐beach unfaithful individuals are thus rarely recorded at the monitored site as they are predominantly using other beaches, with implications for clutch counts and population size estimation (Figure 5).

**FIGURE 5:**
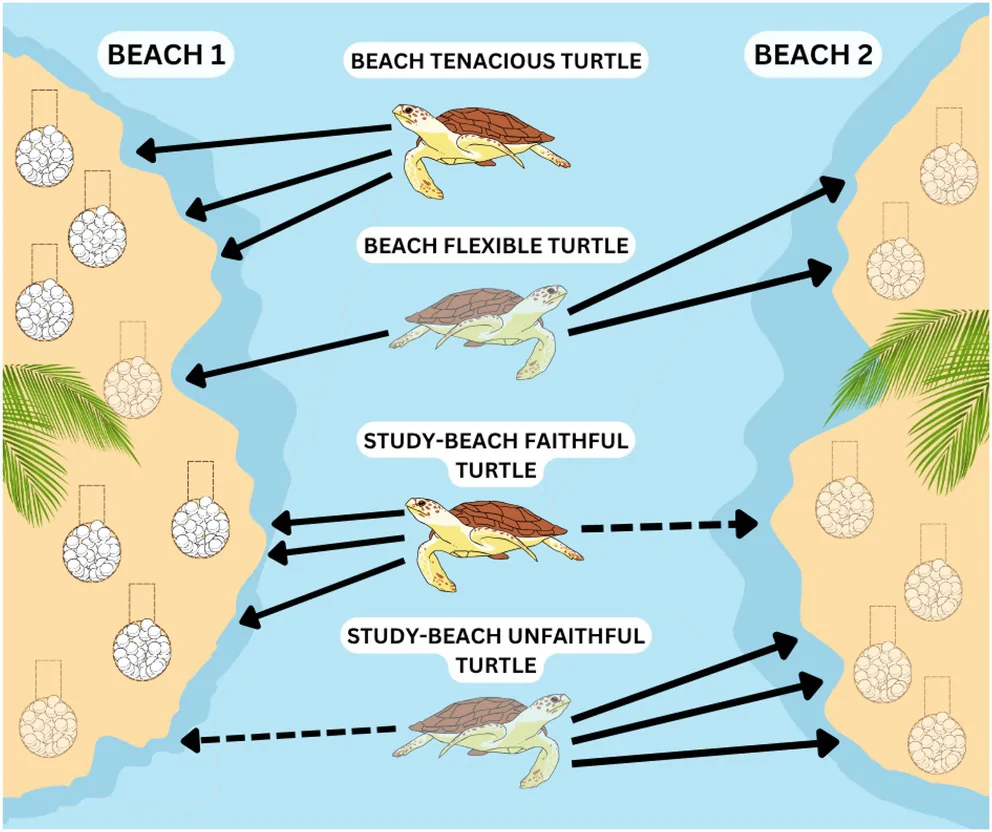
Conceptual diagram illustrating mixed nesting beach fidelity strategies, showing a beach tenacious turtle that is, an individual that lays all of its clutches on beach 1, and a beach flexible turtle, that is, an individual that lays one clutch on beach 1 and lays remaining clutches on multiple beaches within and across seasons. If only beach 1 is monitored, individuals will be observed as study‐beach faithful turtles with respect to beach 1, that is, an individual that lays all or most of its clutches on this primary beach and occasional clutches on a secondary beach, whereas a study‐beach unfaithful turtle with respect to beach 1 is an individual that lays occasional clutches on beach 1 and remaining clutches elsewhere. For single‐site flipper‐tagging programmes, some or most of the clutches of study‐beach unfaithful turtles would be missed, resulting in underestimated clutch frequency and adult female abundance (faded clutches and turtles).

### 3.6 Incidence of Study‐Beach Unfaithful Behaviour Across Sea Turtle Species

Individuals showing study‐beach unfaithful behaviour were reported in the literature for five species at 43 nesting beaches between 1971 and 2026 (*n =* 51 studies; Figure 6 and Table S6). The proportion of study‐beach unfaithful turtles varied across species: 25.6% in loggerhead turtles (0.90%–64%; *n* = 22 studies), followed by 14.5% in leatherbacks (2.82%–53.12%; *n* = 6 studies), 9.3% in green turtles (0.02%–42.55%; *n* = 15 studies), 7.9% in hawksbills (0.39%–22.22%; *n* = 9 studies), and 6.3% in olive ridley turtles (0.43%–17.80%; *n* = 3 studies). The prevalence of study‐beach unfaithful turtles depended on the method used; 14.7% through flipper tagging (range: 0.02%–64%, *n* = 24 studies), 19.4% through satellite tracking (≥ 5 tracked individuals; 3.22%–53.13%, *n* = 14 studies), and 46% through genetic analysis (0.39%–64%, *n* = 2 studies). For island beach studies (*n* = 19 studies), the estimated prevalence of study‐beach unfaithful turtles was 9.3% (0.02%–42.55%), 6.7% on oceanic islands and 11.5% on continental islands, compared to 21.4% at mainland sites (including barrier islands; 0.39%–64%, *n* = 19 studies). The length of coastline monitored per study varied, with surveys spanning 1–15.9 km on islands and 1.6–> 1000 km on mainland sites. The greatest recorded distances between nesting emergences of individual turtles were 2250 km for green turtles ( *Chelonia mydas* ; Le Gall and Hughes 1987), 1849 km for leatherback turtles ( *Dermochelys coriacea* ; Horrocks et al. 2016), 725 km for loggerhead turtles (Stoneburner and Ehrhart 1981) and 320 km for olive ridley turtles ( *Lepidochelys olivacea* ; Tripathy and Pandav 2007). For hawksbill turtles, individuals used multiple beaches within and between seasons, with distances between beaches of up to 300 km (Caribbean; Levasseur et al. 2019). This behaviour has not been reported for two species, flatback ( *Natator depressus* ) and Kemp's ridley turtle ( *Lepidochelys kempii* ).

**FIGURE 6:**
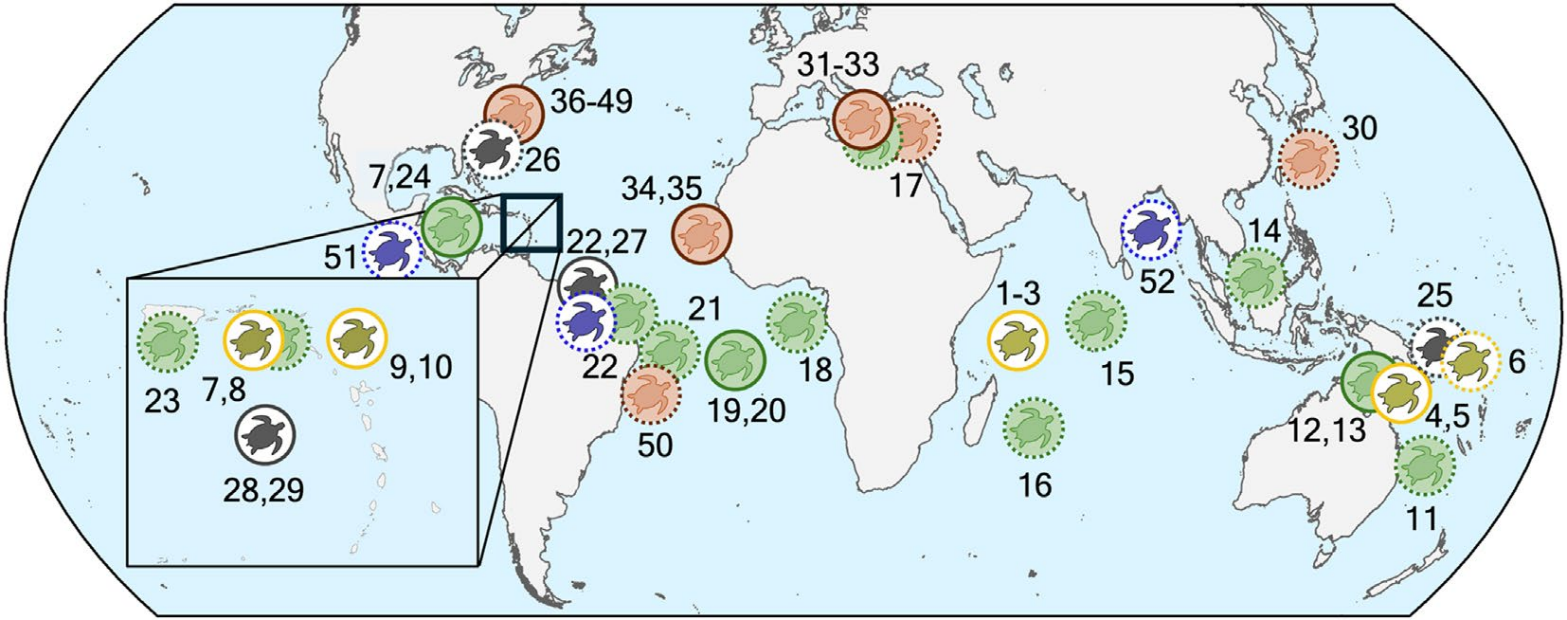
Low nesting fidelity to study beaches was reported at 44 sites around the world (including our current study), that is, individuals recorded laying clutches on multiple beaches for hawksbill turtles (yellow), green turtles (green), leatherback turtles (grey), loggerhead turtles (brown), and olive ridley turtles (blue). Dotted and closed circles indicate single or multiple studies from a region (see Table S6). Hawksbill turtles: 1–3 = Seychelles (present study; Diamond 1976; Allen et al. 2010); 4,5 = Australia (Limpus et al. 2008; Barr et al. 2021); 6 = Solomon Islands (Hamilton et al. 2021); 7–10 = Caribbean Islands (Esteban et al. 2015; Sartain Iverson et al. 2016; Levasseur et al. 2019, 2021). Green turtles: 7 = Caribbean and Costa Rica (Esteban et al. 2015); 11,12,13 = Australia (Dethmers et al. 2006; Hamann et al. 2022; Perez et al. 2024); 14 = Malaysia (Long and Azmi 2017); 15 = Chagos Archipelago (Esteban et al. 2017); 16 = Tromelin (Le Gall and Hughes 1987); 17 = Cyprus (Snape et al. 2018); 18 = Guinea‐Bissau (Raposo et al. 2024); 19,20 = Ascension Island (Mortimer and Portier 1989; Weber et al. 2013); 21 = Brazil (Bellini et al. 2012); 22 = Suriname (Schulz 1971); 23 = Puerto Rico (Kontos et al. 1988); 24 = Costa Rica (Troëng et al. 2005). Leatherback turtles: 25 = Papua New Guinea (Benson et al. 2007); 26 = U.S. (Stewart et al. 2014); 22,27 = Suriname (Schulz 1971; Georges et al. 2007); 28,29 = Caribbean (Horrocks et al. 2016; Eckert et al. 1989). Loggerhead turtles: 17 = Cyprus (Snape et al. 2018); 30 = Japan (Iwamoto et al. 1985); 31–33 = Greece (Hays et al. 1991; Hays and Sutherland 1991; Margaritoulis 1998); 34,35 = Cape Verde (Marco et al. 2011; Martins et al. 2022); 36–49 = Maryland to Florida, U.S. (LeBuff Jr. 1974; Worth and Smith 1976; Bell and Richardson 1978; Talbert Jr. et al. 1980; Stoneburner and Ehrhart 1981; Williams‐Walls et al. 1983; Bjorndal et al. 1983; Hawkes et al. 2005; Lamont and Carthy 2007; Williams and Frick 2008; Tucker 2010; Hart et al. 2013; Shamblin et al. 2017; Pfaller et al. 2022); 50 = Brazil (Marcovaldi et al. 2010). Olive ridley turtles: 51 = Costa Rica (Fusté et al. 2026); 52 = India (Tripathy and Pandav 2007); 22 = Suriname (Schulz 1971).

The percentage of one‐time nesters (individuals recorded nesting only once on a monitored beach) in leatherback and loggerhead turtle populations ranged between 9% and 60% (> 40%, *n* = 4 out of 6 studies; Table S7).

## 4 Discussion

Our study revealed mixed nesting beach fidelity strategies among satellite‐tracked hawksbill turtles on Frégate Island, Seychelles, contributing to growing evidence that fidelity to a single beach is not uniform within turtle populations. We recorded equal proportions of study‐beach faithful and study‐beach unfaithful individuals, with the latter laying clutches across multiple beaches and islands within the same season. Consequently, clutch frequency estimated from foot patrol data was approximately half that derived from satellite tracking. Further, the likelihood of study‐beach unfaithful individuals being resighted on Grand Anse in subsequent seasons was low, as they predominantly nested elsewhere. Globally, our review showed island nesting populations recorded lower proportions of study‐beach unfaithful individuals relative to mainland populations by a factor of 2.4. This pattern likely reflects differences in fidelity strategies, habitat characteristics and monitoring coverage. These findings demonstrate that nesting beach fidelity is a flexible strategy, with important consequences for demographic monitoring and population resilience under climate change.

### 4.1 Hawksbill Beach Fidelity Strategies on Frégate Island

Half of our satellite‐tracked hawksbills were study‐beach faithful, reflecting beach tenacity that likely arises from precise natal philopatry (Lohmann and Lohmann 2019) and has been reported for other hawksbill populations including in Australia (Hoenner et al. 2016). The remaining individuals laid clutches at varying spatial scales: 30% showed island fidelity by using other Frégate beaches, while 20% laid clutches on multiple islands. These findings should be interpreted cautiously as individuals were tracked within a single season, and the proportion of individuals using different fidelity strategies may vary across seasons, nesting cohorts and environmental conditions. Nevertheless, foot patrol records corroborated inter‐beach movements, with at least one satellite‐tagged female using multiple Frégate beaches in 2017–2018, consistent with its beach‐use patterns identified by telemetry in 2023–2024. Although straying from primary beaches is generally associated with neophyte behaviour (Carr et al. 1978; Tucker 2010; Stokes et al. 2014), our findings indicate that even experienced individuals may lay clutches at nearby sites. This may reflect the proximity of suitable alternative beaches to primary nesting sites (Mortimer and Portier 1989; Sartain Iverson et al. 2016; Martins et al. 2022) or represent colonisation of new sites as primary beaches deteriorate or disappear (Carr and Coleman 1974). Further assessment of breeding experience, site proximity and habitat quality will help clarify within‐season movements between beaches.

Study‐beach unfaithful individuals introduce bias into estimates of commonly used population parameters derived from single‐site monitoring. We found that clutch frequency estimated from our satellite tracking data was nearly twice as high as foot patrol estimates, reflecting missed nesting emergences on Frégate and clutches laid on other islands. Similar mismatches between telemetry‐ and foot patrol‐derived estimates have been reported in Florida, at Ascension Island and in Brazil (Tucker 2010; Weber et al. 2013; Santos et al. 2021), highlighting the challenges of incomplete detection. Our results further indicate that study‐beach unfaithful individuals were less likely to be resighted in subsequent seasons, consistent with previous findings (Hays and Sutherland 1991; Stokes et al. 2014). Consequently, parameters dependent on resighting individuals (e.g., remigration rate) are likely underestimated when monitoring efforts target a single location (Shamblin et al. 2021). Combining satellite tracking data with sighting records across regional monitoring programmes can substantially reduce biases arising from mixed beach fidelity strategies and incomplete detection, improving the reliability of demographic estimates.

Estimates of adult female abundance derived from clutch counts assume that observed nests represent the total reproductive output at the study beach. However, this assumption is violated where multiple study‐beach unfaithful individuals lay clutches across multiple beaches that go unaccounted for by single‐site monitoring. Our findings showed that regardless of the clutch frequency, abundance estimates were 2.5 times higher when 40% of clutches were laid on the study beach compared to working on the assumption that all clutches were laid on the same beach. This demonstrates that different fidelity strategies influence abundance estimates independently of clutch frequency. The consequence of incomplete clutch detection at the study beach for adult female abundance estimation, and the importance of quantifying the proportion of clutches laid on other beaches to reduce this bias, have been highlighted previously (Pfaller et al. 2022). Recent studies have introduced approaches that account for imperfect detection due to missed nests or fidelity extending beyond a single beach to provide robust estimates of adult female abundance (Casale et al. 2022). While these approaches improve demographic inference, reliable abundance estimation requires characterising the true extent of fidelity within a subpopulation as this introduces bias that cannot be resolved using clutch frequency alone.

### 4.2 Global Patterns of Sea Turtle Nesting Beach Fidelity

Our review of 51 studies showed that study‐beach unfaithful individuals were 3.2 and 1.8 times more prevalent at mainland than at oceanic and continental island nesting sites respectively, likely attributable at least in part to differences in geomorphology. Mainland beaches are often contiguous with suitable nesting habitat broadly distributed and few physical barriers separating adjacent sites. This may lead females to be less selective of specific sites and more likely to move between beaches (Marcovaldi et al. 2010; Tucker 2010). In contrast, island systems offer spatially limited and patchily distributed habitats that may promote natal philopatry, resulting in strong selection for precise homing to isolated beaches, perhaps enabling navigation with greater precision than to sites along a continuous coastline (Levasseur et al. 2019). Multiple generations of hawksbills returned to a 1‐km beach in Jumby Bay, Antigua, displaying long‐term beach tenacity (Levasseur et al. 2021). At Ascension Island in the South Atlantic where the entire nesting population is restricted to a single island, turtles nevertheless demonstrated strong fidelity to individual beaches or clusters of beaches within the confines of that island (Mortimer and Portier 1989). Patterns may vary among island systems, potentially due to differences in habitat stability. Jumby Bay represents a relatively stable and sheltered nesting environment (Levasseur et al. 2021), whereas Grand Anse on Frégate experiences seasonal erosion during the nesting season, reducing habitat predictability that may result in a higher proportion of beach flexible individuals (J. A. Mortimer pers. comm., 2023). While ECFs of 1.9 and 3.7 were determined at Frégate Island respectively by foot patrols and satellite tracking, elsewhere in Seychelles at Cousin Island, where nesting beaches provide longer, more contiguous and more stable nesting habitat, a minimum ECF of 3.6 clutches was determined based entirely on foot patrol data (Mortimer and Bresson 1999). This contrast suggests that fidelity is modulated by site‐specific characteristics rather than being a fixed trait. Comparative analyses across island systems with varying habitat extent, isolation and temporal variability could test whether fidelity strategies are influenced by environmental conditions.

Monitoring regimes may also play a role in biasing relative levels of documented beach fidelity. We found that beach flexibility has been more frequently documented in loggerhead turtles than in other species, which may reflect differences in monitoring coverage rather than species‐level differences in fidelity behaviour. For example, loggerhead nesting beaches extend 1000s of km across the US and have been surveyed using diverse methods, increasing the probability of detecting beach flexible individuals (Shamblin et al. 2021; Pfaller et al. 2022). By contrast, hawksbill studies are often conducted by project personnel working within the confines of an individual island, with beach patrols that encompass relatively short expanses of nesting beach (Diamond 1976; Allen et al. 2010). The probability of detecting beach flexible individuals is therefore constrained by length of monitored beach relative to the maximum distances between nests. For instance, when satellite telemetry was combined with ground truth patrols of a 6‐km beach in Florida, the mean maximum distance between nests was 26 km for loggerheads, identifying 46% of individuals as beach flexible (Tucker 2010). For islands where patrols are conducted on a single beach, movements to inaccessible beaches and nearby islands will be entirely missed unless regional data are shared between island‐based monitoring programmes. It follows that at locations where population trends are defined by counts of nesting females rather than egg clutches, apparent increases in nesting numbers may partly reflect improved detection of previously undetected nesting females rather than true population growth alone (Pfaller et al. 2013).

### 4.3 Implications of Mixed Fidelity Strategies

The coexistence of beach tenacious and flexible individuals within a population raises questions about the fitness consequences of each strategy under environmental change. Beach tenacity likely confers advantages when local conditions are stable, as individual familiarity with habitat characteristics, resource availability and threat exposure may translate into consistent reproductive success. For example, *Phelsuma* spp. geckos showed oviposition site tenacity because of the advantages of thermally stable laying conditions on Reunion Island (Choeur et al. 2022). Returning to the same site may become maladaptive if habitat quality deteriorates or new threats emerge, creating fidelity‐induced ecological traps (Merkle et al. 2022). For instance, high breeding site fidelity of Baltic eiders reduced adult survival following increased predation by white‐tailed sea eagles (Ekroos et al. 2012), illustrating the costs of beach tenacity when local conditions change unpredictably. For sea turtles, beach tenacity under accelerating coastal erosion and inundation due to sea level rise (Rivas et al. 2023) may reduce reproductive success and compromise long‐term population viability.

Beach flexible individuals, by contrast, may respond better to habitat loss by relocating to alternative beaches and colonising new areas. Spreading clutches across multiple beaches may further serve as a bet‐hedging strategy against localised disturbance by diversifying incubation conditions (Yasui 2022). For example, male poison frogs distributed tadpoles across multiple pools to reduce offspring mortality risk from pool desiccation (Erich et al. 2015). Such dispersal between sites may also improve regional connectivity by maintaining gene flow between subpopulations from different breeding grounds, as recorded for Atlantic herrings (Moll et al. 2025). Although this flexibility may incur costs if the use of alternative sites has unfavourable reproductive consequences (Ward and Weatherhead 2005), the capacity to explore and switch among beaches may prove beneficial as habitats are altered by climate change. At the population level, the prevalence of both behaviours may maintain reproductive output under stable conditions while providing the adaptive capacity to mitigate the impact of habitat loss.

Our study demonstrates that nesting beach fidelity is flexible for a hawksbill subpopulation in the Seychelles, with mixed strategies likely supporting reproductive success under contrasting environmental conditions. Even with high sampling effort at a single site, beach flexible individuals may lead to underestimated mean clutch frequencies and to overestimated adult female abundance. This is compounded by maximum inter‐nest distances that far exceed survey coverage, meaning movements between beaches go undetected. In turn, these biases have the potential to misinform regional population assessments if they are based on estimated numbers of nesting females rather than on clutch counts (Mortimer et al. 2020). Such assessments are crucial for Regional Management Unit (RMU) status (Wallace et al. 2023) and more broadly, Red List conservation assessments (IUCN 2025). The capacity to use multiple beaches may safeguard population longevity as impacts of sea level rise reduce the availability of suitable nesting habitat (Staines et al. 2025). Quantifying the proportion of beach flexible individuals within a population requires coordinating resighting data across regional programmes and where feasible, integrating available satellite telemetry with traditional beach monitoring methods. Comparative studies of island subpopulations nesting under varied habitat stability will help determine whether fidelity plasticity is widespread or a localised response to environmental conditions. Further, temporal assessments at beaches where baseline fidelity strategies have been established will be essential to detect whether fidelity shifts towards beach flexibility as habitats degrade. As nesting cohorts respond to climate change pressures, determining the proportion of beach flexible individuals that may provide population‐level resilience will be useful for prioritising conservation interventions.

## Author Contributions

**Nupur Kale:** data curation (equal), formal analysis (lead), investigation (equal), visualization (lead), writing – original draft (lead), writing – review and editing (lead). **Graeme C. Hays:** conceptualization (lead), formal analysis (supporting), funding acquisition (equal), investigation (equal), methodology (equal), project administration (equal), resources (equal), supervision (equal), validation (equal), writing – original draft (supporting), writing – review and editing (equal). **Jeanne A. Mortimer:** conceptualization (lead), investigation (equal), methodology (equal), validation (equal), writing – review and editing (equal). **Anna Zora:** conceptualization (lead), data curation (equal), funding acquisition (equal), investigation (equal), methodology (equal), project administration (equal), resources (equal), validation (equal), writing – review and editing (equal). **Kimberley L. Stokes:** supervision (equal), validation (equal), writing – review and editing (equal). **Nicole Esteban:** conceptualization (lead), funding acquisition (equal), investigation (equal), methodology (equal), project administration (equal), resources (equal), supervision (equal), validation (equal), writing – original draft (supporting), writing – review and editing (equal).

## Funding

This work was supported by the Bertarelli Foundation as part of the Bertarelli Programme in Marine Science (Grant 820633) and Frégate Island Sanctuary.

## Ethics Statement

All work was approved by the Swansea University Animal Welfare Ethics Review Board (Approval number: 3 2023 7095 7307) and the Seychelles Bureau of Standards (SBS). The study was endorsed through research permits (reference A0157) from the SBS and the research complied with all relevant local and national legislation.

## Conflicts of Interest

The authors declare no conflicts of interest.

## Supporting information


**Table S1:** Residency duration of satellite‐tagged hawkbill turtles.
**Table S2:** Observed and estimated clutch frequencies recorded through foot patrols (2017–2018 to 2024–2025) and satellite tracking (2023–2024).
**Table S3:** Nesting summary of hawksbill turtles satellite tagged on Grand Anse beach, Frégate Island, Seychelles.
**Table S4:** Remigration status of individuals that laid 1 and > 1 clutch in 2017–2018 and 2018–2019.
**Table S5:** Adult female abundance estimated across varying fidelity.
**Table S6:** Global reports of sea turtle species using more than one nesting beach within or between seasons.
**Table S7:** Percentage of one‐time nesting turtles, that is, individuals that nested only once on a particular beach, recorded across sites.

## Data Availability

The dataset supporting this study is included in the Supporting Information file and is available via Dryad Digital Repository https://doi.org/10.5061/dryad.s7h44j1qr.

## References

[ece374341-bib-0001] Allen, Z. C. , N. J. Shah , A. Grant , G. Derand , and D. Bell . 2010. “Hawksbill Turtle Monitoring in Cousin Island Special Reserve, Seychelles: An Eight‐Fold Increase in Annual Nesting Numbers.” Endangered Species Research 11: 195–200. 10.3354/esr00281.

[ece374341-bib-0002] Barr, C. E. , M. Hamann , T. Shimada , I. Bell , C. J. Limpus , and J. Ferguson . 2021. “Post‐Nesting Movements and Feeding Ground Distribution by the Hawksbill Turtle ( *Eretmochelys imbricata* ) From Rookeries in the Torres Strait.” Wildlife Research 48: 598–608. 10.1071/WR20183.

[ece374341-bib-0003] Bell, R. , and J. I. Richardson . 1978. “An Analysis of Tag Recoveries From Loggerhead Sea Turtles ( *Caretta caretta* ) Nesting on Little Cumberland Island, Georgia.” Florida Marine Research Publications 33: 20–24. http://www.seaturtle.org/PDF/HendersonGE_1978_FMRTechReport.pdf#page=27.

[ece374341-bib-0004] Bellini, C. , A. J. B. Santos , A. Grossman , M. A. Marcovaldi , and P. C. R. Barata . 2012. “Green Turtle ( *Chelonia mydas* ) Nesting on Atol das Rocas, North‐Eastern Brazil, 1990–2008.” Journal of the Marine Biological Association of the United Kingdom 93: 1117–1132. 10.1017/S002531541200046X.

[ece374341-bib-0005] Benson, S. R. , K. M. Kisokau , L. Ambio , V. Rei , P. H. Dutton , and D. Parker . 2007. “Beach Use, Internesting Movement, and Migration of Leatherback Turtles, *Dermochelys coriacea* , Nesting on the North Coast of Papua New Guinea.” Chelonian Conservation and Biology 6: 7–14. 10.2744/1071-8443(2007)6[7:BUIMAM]2.0.CO;2.

[ece374341-bib-0006] Berthold, P. , A. J. Helbig , G. Mohr , and U. Querner . 1992. “Rapid Microevolution of Migratory Behaviour in a Wild Bird Species.” Nature 360: 668–670. 10.1038/360668a0.

[ece374341-bib-0007] Bjorndal, K. A. , A. B. Meylan , and B. J. Turner . 1983. “Sea Turtles Nesting at Melbourne Beach, Florida, I. Size, Growth and Reproductive Biology.” Biological Conservation 26: 65–77. 10.1016/0006-3207(83)90049-6.

[ece374341-bib-0008] Bolten, A. B. 1999. “Techniques for Measuring Sea Turtles.” In Research and Management Techniques for the Conservation of Sea Turtles, edited by K. L. Eckert , K. A. Bjorndal , F. A. Abreu‐Grobois , and M. Donnelly , vol. 4, 110–114. IUCN/SSC Marine Turtle Specialist Group.

[ece374341-bib-0009] Carr, A. 1975. “The Ascension Island Green Turtle Colony.” Copeia 1975: 547–555. 10.2307/1443656.

[ece374341-bib-0010] Carr, A. , and P. J. Coleman . 1974. “Seafloor Spreading Theory and the Odyssey of the Green Turtle.” Nature 249: 128–130. 10.1038/249128a0.

[ece374341-bib-0011] Carr, A. F. , M. H. Carr , and A. B. Meylan . 1978. “The Ecology and Migrations of Sea Turtles 7: The West Caribbean Green Turtle Colony.” Bulletin of the American Museum of Natural History 162: 1–48. http://hdl.handle.net/2246/1287.

[ece374341-bib-0012] Casale, P. , S. A. Ceriani , M. G. Dodd , et al. 2022. “New Methods to Derive Sea Turtle Nester Abundance From Nest Counts: Ground Truthing and the Bias of Current Approaches.” Biological Conservation 275: 109754. 10.1016/j.biocon.2022.109754.

[ece374341-bib-0013] Ceriani, S. A. , P. Casale , M. Brost , E. H. Leone , and B. E. Witherington . 2019. “Conservation Implications of Sea Turtle Nesting Trends: Elusive Recovery of a Globally Important Loggerhead Population.” Ecosphere 10: e02936. 10.1002/ecs2.2936.

[ece374341-bib-0014] Choeur, A. , J. Clémencet , M. Le Corre , and M. Sanchez . 2022. “Evidence of Seasonal Reproduction, Laying Site Fidelity, and Oviposition Synchronicity in the Critically Endangered Endemic Manapany Day Gecko ( *Phelsuma inexpectata* ) From Reunion Island (Western Indian Ocean).” Salamandra 58: 116–122. https://archimer.ifremer.fr/doc/00822/93414/.

[ece374341-bib-0015] Dethmers, K. E. M. , D. Broderick , C. Moritz , et al. 2006. “The Genetic Structure of Australasian Green Turtles ( *Chelonia mydas* ): Exploring the Geographical Scale of Genetic Exchange.” Molecular Ecology 15: 3931–3946. 10.1111/j.1365-294X.2006.03070.x. 17054494

[ece374341-bib-0016] Diamond, A. W. 1976. “Breeding Biology and Conservation of Hawksbill Turtles, *Eretmochelys imbricata* L., on Cousin Island, Seychelles.” Biological Conservation 9: 199–215. 10.1016/0006-3207(76)90010-0.

[ece374341-bib-0017] Dujon, A. M. , T. R. Lindstrom , and G. C. Hays . 2014. “The Accuracy of Fastloc‐GPS Locations and Implications for Animal Tracking.” Methods in Ecology and Evolution 5: 1162–1169. 10.1111/2041-210X.12286.

[ece374341-bib-0018] Eckert, K. L. , S. A. Eckert , T. W. Adams , and A. D. Tucker . 1989. “Inter‐Nesting Migrations by Leatherback Sea Turtles ( *Dermochelys coriacea* ) in the West Indies.” Herpetologica 45: 190–194. https://www.jstor.org/stable/3892161.

[ece374341-bib-0019] Ekroos, J. , M. Öst , P. Karell , K. Jaatinen , and M. Kilpi . 2012. “Philopatric Predisposition to Predation‐Induced Ecological Traps: Habitat‐Dependent Mortality of Breeding Eiders.” Oecologia 170: 979–986. 10.1007/s00442-012-2378-9. 22700063

[ece374341-bib-0020] Erich, M. , M. Ringler , W. Hödl , and E. Ringler . 2015. “Brood‐Partitioning Behaviour in Unpredictable Environments: Hedging the Bets?” Behavioural Ecology & Sociobiology 69: 1011–1017. 10.1007/s00265-015-1913-1. PMC4425806 25983381

[ece374341-bib-0021] Esteban, N. , J. A. Mortimer , and G. C. Hays . 2017. “How Number of Nesting Sea Turtles Can Be Overestimated by Nearly a Factor of Two.” Proceedings of the Royal Society B: Biological Sciences 284: 20162581. 10.1098/rspb.2016.2581. PMC5326529 28202810

[ece374341-bib-0022] Esteban, N. , R. P. van Dam , E. Harrison , A. Herrera , and J. Berkel . 2015. “Green and Hawksbill Turtles in the Lesser Antilles Demonstrate Behavioural Plasticity in Inter‐Nesting Behaviour and Post‐Nesting Migration.” Marine Biology 162: 1153–1163. 10.1007/s00227-015-2656-2.

[ece374341-bib-0023] Fagan, W. F. , M. A. Lewis , M. Auger‐Méthe , et al. 2013. “Spatial Memory and Animal Movement.” Ecology Letters 16: 316–1329. 10.1111/ele.12165. 23953128

[ece374341-bib-0024] Fusté, R. , C. Mejías‐Balsalobre , E. G. Webster , I. Naranjo , R. Arauz , and D. Rojas‐Cañizales . 2026. “Inter‐Beach Movements and Inter‐Nesting Periods of Olive Ridley Sea Turtles Relate to Nesting Behavior in a Nascent Arribada Beach.” Marine Biology 173: 117. 10.1007/s00227-026-04856-z.

[ece374341-bib-0025] GEBCO Compilation Group . 2026. “GEBCO 2026 Grid.” 10.5285/4f68d5c7-45eb-f999-e063-7086abc036fa.

[ece374341-bib-0026] Gehr, B. , N. C. Bonnot , M. Heurich , et al. 2020. “Stay Home, Stay Safe‐Site Familiarity Reduces Predation Risk in a Large Herbivore in Two Contrasting Study Sites.” Journal of Animal Ecology 89: 1329–1339. 10.1111/1365-2656.13202. 32144759

[ece374341-bib-0027] Georges, J.‐Y. , S. Fossette , A. Billes , et al. 2007. “Meta‐Analysis of Movements in Atlantic Leatherback Turtles During the Nesting Season: Conservation Implications.” Marine Ecology Progress Series 338: 225–232. 10.3354/meps338225.

[ece374341-bib-0028] Gerber, B. D. , M. B. Hooten , C. P. Peck , et al. 2019. “Extreme Site Fidelity as an Optimal Strategy in an Unpredictable and Homogenous Environment.” Functional Ecology 33: 1695–1707. 10.1111/1365-2435.13390.

[ece374341-bib-0029] Hamann, M. , T. Shimada , S. Duce , A. Foster , A. Tsz Ying To , and C. Limpus . 2022. “Patterns of Nesting Behaviour and Nesting Success for Green Turtles at Raine Island, Australia.” Endangered Species Research 47: 217–229. 10.3354/esr01175.

[ece374341-bib-0030] Hamilton, R. J. , A. Desbiens , J. Pita , et al. 2021. “Satellite Tracking Improves Conservation Outcomes for Nesting Hawksbill Turtles in Solomon Islands.” Biological Conservation 261: 109240. 10.1016/j.biocon.2021.109240.

[ece374341-bib-0031] Hart, K. M. , M. M. Lamont , A. R. Sartain , I. Fujisaki , and B. S. Stephens . 2013. “Movements and Habitat‐Use of Loggerhead Sea Turtles in the Northern Gulf of Mexico During the Reproductive Period.” PLoS One 8: e66921. 10.1371/journal.pone.0066921. PMC3700946 23843971

[ece374341-bib-0032] Hawkes, L. A. , A. C. Broderick , M. H. Godfrey , and B. J. Godley . 2005. “Status of Nesting Loggerhead Turtles, *Caretta caretta* , at Bald Head Island (North Carolina, USA) After 24 Years of Intensive Monitoring and Conservation.” Oryx 39: 65–72. 10.1017/S0030605305000116.

[ece374341-bib-0033] Hays, G. C. , and J. M. Sutherland . 1991. “Remigration and Beach Fidelity of Loggerhead Turtles Nesting on the Island of Cephalonia, Greece.” Journal of Herpetology 25: 232–233. https://www.jstor.org/stable/1564660.

[ece374341-bib-0034] Hays, G. C. , P. I. Webb , J. P. Hayes , I. G. Priede , and J. French . 1991. “Satellite Tracking of a Loggerhead Turtle ( *Caretta caretta* ) in the Mediterranean.” Journal of the Marine Biological Association of the United Kingdom 71: 743–746. 10.1017/S0025315400053261.

[ece374341-bib-0035] Hoenner, X. , S. D. Whiting , G. Enever , K. Lambert , M. A. Hindell , and C. R. McMahon . 2016. “Nesting Ecology of Hawksbill Turtles at a Rookery of International Significance in Australia's Northern Territory.” Wildlife Research 43: 461–473. 10.1071/WR16047.

[ece374341-bib-0036] Horrocks, J. A. , S. Stapleton , H. Guada , et al. 2016. “International Movements of Adult Female Leatherback Turtles in the Caribbean: Results From Tag Recovery Data (2002‐2013).” Endangered Species Research 29: 279–287. 10.3354/esr00718.

[ece374341-bib-0037] IUCN . 2025. “The IUCN Red List of Threatened Species.” Version 2025‐1. Accessed July 16, 2025. https://www.iucnredlist.org.

[ece374341-bib-0038] Iwamoto, T. , M. Ishii , Y. Nakashima , H. Takeshita , and A. Itoh . 1985. “Nesting Cycles and Migrations of the Loggerhead Sea Turtle in Miyazaki, Japan.” Japanese Journal of Ecology 35: 505–511. 10.18960/seitai.35.4_505.

[ece374341-bib-0039] Johnson, S. A. , and L. M. Ehrhart . 1996. “Reproductive Ecology of the Florida Green Turtle: Clutch Frequency.” Journal of Herpetology 30: 407–410. 10.2307/1565180.

[ece374341-bib-0040] Kontos, A. , S. Eckert , K. Eckert , J. Gomez , R. Lee , and R. Van Dam . 1988. “Inter‐Island Migration of Nesting Green Turtle, *Chelonia mydas* .” Marine Turtle Newsletter 42: 10–11.

[ece374341-bib-0041] Kreling, S. E. S. , K. M. Gaynor , A. McInturff , K. L. Calhoun , and J. S. Brashares . 2021. “Site Fidelity and Behavioral Plasticity Regulate an Ungulate's Response to Extreme Disturbance.” Ecology and Evolution 11: 15683–15694. 10.1002/ece3.8221. PMC8601917 34824782

[ece374341-bib-0042] Lafontaine, A. , P. Drapeau , D. Fortin , and M.‐H. St‐Laurent . 2017. “Many Places Called Home: The Adaptive Value of Seasonal Adjustments in Range Fidelity.” Journal of Animal Ecology 86: 624–633. 10.1111/1365-2656.12645. 28146328

[ece374341-bib-0043] Lamont, M. M. , and R. R. Carthy . 2007. “Response of Nesting Sea Turtles to Barrier Island Dynamics.” Chelonian Conservation and Biology 6: 206–212. 10.2744/1071-8443(2007)6[206:RONSTT]2.0.CO;2.

[ece374341-bib-0044] Le Gall, J.‐Y. , and G. R. Hughes . 1987. “Migrations de la tortue verte *Chelonia mydas* dans l'Ocean Indien Sud‐Ouest observees a partir des marquages sur les de ponte Europa et Tromelin (1970–1985).” Amphibia‐Reptilia 8: 277–282. 10.1163/156853887X00315.

[ece374341-bib-0045] LeBuff, C. R., Jr. 1974. “Unusual Nesting Relocation in the Loggerhead Turtle, *Caretta caretta* .” Herpetologica 30: 29–31.

[ece374341-bib-0046] Levasseur, K. E. , S. P. Stapleton , M. C. Fuller , and J. M. Quattro . 2019. “Exceptionally High Natal Homing Precision in Hawksbill Sea Turtles to Insular Rookeries of the Caribbean.” Marine Ecology Progress Series 620: 155–171. 10.3354/meps12957.

[ece374341-bib-0047] Levasseur, K. E. , S. P. Stapleton , and J. M. Quattro . 2021. “Precise Natal Homing and an Estimate of Age at Sexual Maturity in Hawksbill Turtles.” Animal Conservation 24: 523–535. 10.1111/acv.12657.

[ece374341-bib-0048] Limpus, C. J. , J. D. Miller , M. Guinea , and S. Whiting . 2008. Australian Hawksbill Turtle Population Dynamics Project, 140. Environmental Protection Agency.

[ece374341-bib-0049] Lohmann, K. J. , and C. M. Lohmann . 2019. “There and Back Again: Natal Homing by Magnetic Navigation in Sea Turtles and Salmon.” Journal of Experimental Biology 222: jeb184077. 10.1242/jeb.184077. 30728225

[ece374341-bib-0050] Long, S.‐L. , and N. A. Azmi . 2017. “Using Photographic Identification to Monitor Sea Turtle Populations at Perhentian Islands Marine Park in Malaysia.” Herpetological Conservation and Biology 12: 350–366.

[ece374341-bib-0051] Marco, A. , E. A. Pérez , C. M. Argüello , S. Martins , S. Araujo , and L. F. L. Jurado . 2011. “The International Importance of the Archipelago of Cape Verde for Marine Turtles, in Particular the Loggerhead Turtle *Caretta caretta* .” Zoologia Caboverdiana 2: 1–11. http://hdl.handle.net/10553/18267.

[ece374341-bib-0052] Marcovaldi, M. A. , G. G. Lopez , L. S. Soares , E. H. S. M. Lima , J. C. A. Thomé , and A. P. Almeida . 2010. “Satellite‐Tracking of Female Loggerhead Turtles Highlights Fidelity Behaviour in Northeastern Brazil.” Endangered Species Research 12: 263–272. 10.3354/esr00308.

[ece374341-bib-0053] Margaritoulis, D. 1998. “Interchange of Nesting Loggerheads Among Greek Beaches.” In Proceedings of the Seventeenth Annual Sea Turtle Symposium, edited by S. P. Epperly and J. Braun . NOAA Technical Memorandum NMPS‐SEFC‐415.

[ece374341-bib-0054] Martins, S. , L. Cardona , E. Abella , et al. 2022. “Effect of Body Size on the Long‐Term Reproductive Output of Eastern Atlantic Loggerhead Turtles *Caretta caretta* .” Endangered Species Research 48: 175–189. 10.3354/esr01198.

[ece374341-bib-0055] Merkle, J. A. , B. Abrahms , J. B. Armstrong , H. Sawyer , D. P. Costa , and A. D. Chalfoun . 2022. “Site Fidelity as a Maladaptive Behavior in the Anthropocene.” Frontiers in Ecology & Environment 20: 187–194. 10.1002/fee.2456.

[ece374341-bib-0056] Moll, D. , P. Polte , K. P. Jochum , et al. 2025. “Direct Evidence of Natal Homing in an Atlantic Herring Metapopulation.” Science Advances 11: eadz6746. 10.1126/sciadv.adz6746. PMC12577671 41171912

[ece374341-bib-0057] Mortimer, J. A. , and R. Bresson . 1999. “Temporal Distribution and Periodicity in Hawksbill Turtles ( *Eretmochelys imbricata* ) Nesting at Cousin Island, Republic of Seychelles, 1971–1997.” Chelonian Conservation and Biology 3: 292–298.

[ece374341-bib-0058] Mortimer, J. A. , N. Esteban , A. N. Guzman , and G. C. Hays . 2020. “Estimates of Marine Turtle Nesting Populations in the South‐West Indian Ocean Indicate the Importance of the Chagos Archipelago.” Oryx 54: 332–343. 10.1017/S0030605319001108.

[ece374341-bib-0059] Mortimer, J. A. , and K. M. Portier . 1989. “Reproductive Homing and Interesting Behavior of the Green Turtle ( *Chelonia mydas* ) at Ascension Island, South Atlantic Ocean.” Copeia 1989: 962–977. 10.2307/1445982.

[ece374341-bib-0060] Perez, M. , C. Limpus , T. Shimada , et al. 2024. “Inter‐Nesting Habitat Use by Green Turtles *Chelonia mydas* in the Great Barrier Reef.” Endangered Species Research 54: 225–238. 10.3354/esr01340.

[ece374341-bib-0061] Pfaller, J. B. , K. A. Bjorndal , M. Chaloupka , K. L. Williams , M. G. Frick , and A. B. Bolten . 2013. “Accounting for Imperfect Detection Is Critical for Inferring Marine Turtle Nesting Population Trends.” PLoS One 8: e62326. 10.1371/journal.pone.0062326. PMC3634727 23638041

[ece374341-bib-0062] Pfaller, J. B. , S. M. Weaver , K. L. Williams , et al. 2022. “One Beach Amongst Many: How Weak Fidelity to a Focal Nesting Site Can Bias Demographic Rates in Marine Turtles.” Marine Biology 169: 8. 10.1007/s00227-021-03991-z.

[ece374341-bib-0063] R Core Team . 2026. R: A Language and Environment for Statistical Computing. R Foundation for Statistical Computing. https://www.R‐project.org/.

[ece374341-bib-0064] Ramellini, S. , S. Imperio , J. Morinay , et al. 2022. “Individual Foraging Site Fidelity Increases From Incubation to Nestling Rearing in a Colonial Bird.” Animal Behaviour 193: 145–155. 10.1016/j.anbehav.2022.07.014.

[ece374341-bib-0065] Raposo, C. , R. Rebelo , P. Catry , et al. 2024. “Inter‐Island Nesting Dynamics and Clutch Survival of Green Turtles *Chelonia mydas* Within a Marine Protected Area in the Bijagós Archipelago, West Africa.” Marine Biology 171: 143. 10.1007/s00227-024-04463-w.

[ece374341-bib-0066] Richardson, J. I. 1982. A Population Model for Adult Female Loggerhead Sea Turtles ( *Caretta caretta* ) Nesting in Georgia. PhD Dissertation. University of Georgia.

[ece374341-bib-0067] Rivas, M. L. , E. Rodríguez‐Caballero , N. Esteban , et al. 2023. “Uncertain Future for Global Sea Turtle Populations in Face of Sea Level Rise.” Scientific Reports 13: 5277. 10.1038/s41598-023-31467-1. PMC10119306 37081050

[ece374341-bib-0068] Santos, A. J. B. , D. H. G. Vieira , C. Bellini , G. Corso , S. A. Ceriani , and M. M. P. B. Fuentes . 2021. “Using Data From Nesting Beach Monitoring and Satellite Telemetry to Improve Estimates of Marine Turtle Clutch Frequency and Population Abundance.” Marine Biology 168: 170. 10.1007/s00227-021-03983-z.

[ece374341-bib-0069] Sartain Iverson, A. R. , K. M. Hart , I. Fujisaki , et al. 2016. “Hawksbill Satellite‐Tracking Case Study: Implications for Remigration Interval and Population Estimates.” Marine Turtle Newsletter 148: 2–7.

[ece374341-bib-0070] Schmidt, K. A. 2014. “Site Fidelity Curbs Sequential Search and Territory Choice: A Game Theoretical Approach.” Functional Ecology 28: 1494–1503. 10.1111/1365-2435.12291.

[ece374341-bib-0071] Schulz, J. P. 1971. “Situation Report on Marine Turtles Nesting in Surinam.” In Marine Turtles. IUCN Publications New Series, Supplementary Paper 31, 68–74. International Union for Conservation of Nature and Natural Resources.

[ece374341-bib-0072] Shamblin, B. M. , M. G. Dodd , D. B. Griffin , et al. 2017. “Improved Female Abundance and Reproductive Parameter Estimates Through Subpopulation‐Scale Genetic Capture‐Recapture of Loggerhead Turtles.” Marine Biology 164: 138. 10.1007/s00227-017-3166-1.

[ece374341-bib-0073] Shamblin, B. M. , M. G. Dodd , S. M. Pate , et al. 2021. “In Search of the “Missing Majority” of Nesting Loggerhead Turtles: Improved Inter‐Seasonal Recapture Rates Through Subpopulation‐Scale Genetic Tagging.” Marine Biology 168: 16. 10.1007/s00227-020-03820-9.

[ece374341-bib-0074] Shimada, T. , C. J. Limpus , M. Hamann , et al. 2020. “Fidelity to Foraging Sites After Long Migrations.” Journal of Animal Ecology 89: 1008–1016. 10.1111/1365-2656.13157. 31785174

[ece374341-bib-0075] Smith, S. N. , M. D. Jones , B. M. Marshall , S. Waengsothorn , G. A. Gale , and C. T. Strine . 2021. “Native Burmese Pythons Exhibit Site Fidelity and Preference for Aquatic Habitats in an Agricultural Mosaic.” Scientific Reports 11: 7014. 10.1038/s41598-021-86640-1. PMC8007826 33782524

[ece374341-bib-0076] Snape, R. T. E. , P. J. Bradshaw , A. C. Broderick , W. J. Fuller , K. L. Stokes , and B. J. Godley . 2018. “Off‐the‐Shelf GPS Technology to Inform Marine Protected Areas for Marine Turtles.” Biological Conservation 227: 301–309. 10.1016/j.biocon.2018.09.029.

[ece374341-bib-0077] Staines, M. N. , D. T. Booth , J.‐O. Laloë , and G. C. Hays . 2025. “The Impact of Sea Level Rise on Nesting Sea Turtles: A Global Meta‐Analysis to Highlight Current Understanding and Knowledge Gaps.” Marine Biology 172: 171. 10.1007/s00227-025-04726-0.

[ece374341-bib-0078] Stewart, K. R. , K. J. Martin , C. Johnson , N. Desjardin , S. A. Eckert , and L. B. Crowder . 2014. “Increased Nesting, Good Survival and Variable Site Fidelity for Leatherback Turtles in Florida, USA.” Biological Conservation 176: 117–125. 10.1016/j.biocon.2014.05.008.

[ece374341-bib-0079] Stokes, K. L. , W. J. Fuller , F. Glen , et al. 2014. “Detecting Green Shoots of Recovery: The Importance of Long‐Term Individual‐Based Monitoring of Marine Turtles.” Animal Conservation 17: 593–602. 10.1111/acv.12128.

[ece374341-bib-0080] Stoneburner, D. L. , and L. M. Ehrhart . 1981. “Observations on *Caretta c. caretta*: A Record Internesting Migration in the Atlantic.” Herpetological Review 12: 66.

[ece374341-bib-0081] Switzer, P. V. 1993. “Site Fidelity in Predictable and Unpredictable Habitats.” Evolutionary Ecology 7: 533–555. 10.1007/BF01237820.

[ece374341-bib-0082] Talbert, O. R., Jr. , S. E. Stancky , J. M. Dean , and J. M. Will . 1980. “Nesting Activity of the Loggerhead Turtle ( *Caretta caretta* ) in South Carolina I: A Rookery in Transition.” Copeia 1980: 709–718. https://www.jstor.org/stable/1444448.

[ece374341-bib-0083] Tripathy, B. , and B. Pandav . 2007. “Beach Fidelity and Internesting Movements of Olive Ridley Turtles ( *Lepidochelys olivacea* ) at Rushikulya, India.” Herpetological Conservation and Biology 3: 40–45. http://www.seaturtle.org/PDF/TripathyB_2008_HerpConservBiol.pdf.

[ece374341-bib-0084] Troëng, S. , D. R. Evans , E. Harrison , and C. J. Lageaux . 2005. “Migration of Green Turtles From Tortuguero, Costa Rica.” Marine Biology 148: 435–447. 10.1007/s00227-005-0076-4.

[ece374341-bib-0085] Tucker, A. D. 2010. “Nest Site Fidelity and Clutch Frequency of Loggerhead Turtles Are Better Elucidated by Satellite Telemetry Than by Nocturnal Tagging Efforts: Implications for Stock Estimation.” Journal of Experimental Marine Biology and Ecology 383: 48–55. 10.1016/j.jembe.2009.11.009.

[ece374341-bib-0086] Wallace, B. P. , Z. A. Posnik , B. J. Hurley , et al. 2023. “Marine Turtle Regional Management Units 2.0: An Updated Framework for Conservation and Research of Wide‐Ranging Megafauna Species.” Endangered Species Research 52: 209–223. 10.3354/esr01243.

[ece374341-bib-0087] Ward, M. P. , and P. J. Weatherhead . 2005. “Sex‐Specific Difference in Site Fidelity and the Cost of Dispersal in Yellow‐Headed Blackbirds.” Behavioral Ecology and Sociobiology 59: 108114. 10.1007/s00265-005-0015-x.

[ece374341-bib-0088] Weber, N. , S. B. Weber , B. J. Godley , J. Ellick , M. Witt , and A. C. Broderick . 2013. “Telemetry as a Tool for Improving Estimates of Marine Turtle Abundance.” Biological Conservation 167: 90–96. 10.1016/j.biocon.2013.07.030.

[ece374341-bib-0089] Weishampel, J. F. , D. A. Bagley , L. M. Ehrhart , and B. L. Rodenbeck . 2003. “Spatiotemporal Patterns of Annual Sea Turtle Nesting Behaviors Along an East Central Florida Beach.” Biological Conservation 110: 295–303. 10.1016/S0006-3207(02)00232-X.

[ece374341-bib-0090] Williams, K. L. , and M. G. Frick . 2008. “Tag Returns From Loggerhead Turtles From Wassaw Island, GA.” Southeastern Naturalist 7: 165–172. www.jstor.com/stable/20203982.

[ece374341-bib-0091] Williams‐Walls, N. , J. O'Hara , R. M. Gallagher , D. F. Worth , B. D. Peery , and J. R. Wilcox . 1983. “Spatial and Temporal Trends of Sea Turtle Nesting on Hutchinson Island, Florida, 1971‐1979.” Bulletin of Marine Science 33: 55–66.

[ece374341-bib-0092] Worth, D. F. , and J. B. Smith . 1976. “Marine Turtle Nesting on Hutchinson Island, Florida, in 1973.” Florida Marine Research Publications 18: 1–14.

[ece374341-bib-0093] Yasui, Y. 2022. “Evolutionary Bet‐Hedging Reconsidered: What Is the Mean‐Variance Trade‐Off of Fitness?” Ecological Research 37: 406–420. 10.1111/1440-1703.12303.

