# Supplementary material for: Sea Turtle Nesting Beach Fidelity Revealed by Satellite Tracking
Source: Ecol Evol. 2026 Oct 6;16(10):e74341. doi: 10.1002/ece3.74341 (PMC13640060; doi:10.1002/ece3.74341)
Supplement: Supplementary file 1 [file ECE3-16-e74341-s001.pdf]

Sea turtle nesting beach fidelity revealed by satellite tracking

Nupur Kale[1], Graeme C. Hays[2], Jeanne A. Mortimer[3,4], Anna Zora[5], Kimberley L. Stokes[1] & Nicole Esteban[1]*

[1] Department of Biosciences, Swansea University, Singleton Park, Wales, UK

[2] Deakin Marine Research and Innovation Centre, School of Life and Environmental Sciences, Deakin University, Geelong, Victoria, Australia

[3] Victoria, Mahé, Republic of Seychelles

[4] Department of Biology, University of Florida, Gainesville, FL, USA

[5] Frégate Island Foundation, Angelfish Marina, Roche Caiman, Mahé, Republic of Seychelles

*Corresponding author: n.esteban@swansea.ac.uk

Supporting information

Table S1 Residency duration of satellite-tagged hawkbill turtles.

Table S2 Observed and estimated clutch frequencies recorded through foot patrols (2017-18 to 2024-25) and satellite tracking (2023-24).

Table S3 Nesting summary of hawksbill turtles satellite tagged on Grand Anse beach, Frégate Island, Seychelles.

Table S4 Remigration status of individuals that laid 1 and > 1 clutch in 2017-18 and 2018-19.

Table S5 Adult female abundance estimated across varying fidelity.

Table S6 Global reports of sea turtle species using more than one nesting beach within or between seasons.

Table S7 Percentage of one-time nesting turtles, i.e. individuals that nested only once on a particular beach, recorded across sites.

Table S1 Residency at breeding grounds of hawkbill turtles (*Eretmochelys imbricata*) satellite-tagged on Grand Anse, Frégate Island, Seychelles was calculated as the days elapsed between the first observed nesting in 2023-24 and departure from breeding grounds.

**Turtle ID**	**Dates**				**Residency at breeding grounds (d)**
	**First recorded nest in 2023**	**Tag deployment**	**Last recorded nest**	**Departed for foraging grounds**	
250788	30-11-2023	30-11-2023	30-11-2023	02-12-2023	3
250789	31-10-2023	30-11-2023	15-12-2023	16-12-2023	45
250790	01-12-2023	01-12-2023	16-01-2024	17-01-2024	46
250791	01-12-2023	01-12-2023	11-01-2024	11-01-2024	41
250792	19-11-2023	02-12-2023	15-12-2023	16-12-2023	26
250793	03-12-2023	03-12-2023	25-01-2024	26-01-2024	53
250794	30-10-2023	07-12-2023	20-12-2023	20-12-2023	50
250795	08-12-2023	08-12-2023	21-12-2023	22-12-2023	13
250796	02-12-2023	02-12-2023	13-01-2024	14-01-2024	42
250797	04-11-2023	02-12-2023	28-12-2023	29-12-2023	54

Table S2 Clutch frequency of hawksbill turtles (*Eretmochelys imbricata*) estimated from satellite tracking data (2023-24) was higher than clutch frequency derived from foot patrol data across years (2017-18 to 2024-25) on Frégate Island, Seychelles.

**Nesting season**	**Number of individuals (*n*)**	**Mean clutch frequency (SD)**	
		**Observed**	**Estimated**
2017-18	36	1.33 (0.76)	1.47 (1.08)
2018-19	27	1.35 (0.56)	1.73 (1.12)
2019-20	120	1.71 (0.90)	2.08 (1.35)
2020-21	96	1.55 (0.78)	1.91 (1.32)
2021-22	54	2.04 (1.16)	2.52 (1.59)
2022-23	70	1.56 (0.83)	1.79 (1.14)
2023-24	74	1.68 (0.89)	1.97 (1.25)
2024-25	98	1.62 (0.81)	1.98 (1.31)
**Satellite-tracking**	10	NA	3.66 (1.25)

Table S3 Summary of nest locations and nesting duration for hawksbill turtles (*Eretmochelys imbricata*, n = 10) satellite-tagged on Grand Anse beach, Frégate Island, Republic of Seychelles. Nesting events in 2023 and 2024 were identified using: i) haul-out information (> 20 mins; H), ii) filtered Fastloc-GPS locations (F) and iii) foot patrols (P). Times given are local to Seychelles (GMT + 4h). A single asterisk indicates no subsequent nests, and two asterisks indicate nests on other islands.

Turtle ID	CCL (cm)	Days spent at breeding grounds post tag deployment (d)	Nests recorded (*n*)	Nest date (h)	Evidence	Nesting beach	Straight-line distance between first and subsequent nests (km)
250788	86	3	1	30 Nov (13:12)		Grand Anse *	--
250789	92	16	1	30 Nov (15:30)		Grand Anse	--
			1	15 Dec (20:05)	H F	Marina	1.43
250790	83	46	1	1 Dec (13:35)		Grand Anse	--
			1	18 Dec (15:45)	H F	Grand Anse	0.02
			1	1 Jan (17:14)	H F	Grand Anse	0.09
			1	16 Jan (09:46)	H	Anse Parc	1.33
250791	89	41	1	1 Dec (13:50)		Grand Anse	--
			1	16 Dec (08:57)	H F P	Anse Victorin	0.83
			1	28 Dec (13:19)	H F	Anse Macquereau	1.00

Turtle ID	CCL (cm)	Days spent at breeding grounds post tag deployment (d)	Nests recorded (*n*)	Nest date (h)	Evidence	Nesting beach	Straight-line distance between first and subsequent nests (km)
			1	11 Jan (12:03)	H	Anse Bambous	1.20
250792	90	14	1	2 Dec (15:14)		Grand Anse	--
			1	15 Dec (19:02)	H F	Grand Anse	0.17
250793	86	53	1	3 Dec (14:10)		Grand Anse	--
			1	17 Dec (10:09)	H F	Marianne**	27.30
			1	30 Dec (09:06)	H F	Marianne**	27.30
			1	13 Jan (09:00)	H F	Marianne**	27.30
			1	25 Jan (10:00)	H F	Marianne**	27.30
250794	84	13	1	7 Dec (13:30)		Grand Anse	--
			1	20 Dec (08:16)	H F	Grand Anse	0
250795	84	13	1	8 Dec (10:00)		Grand Anse	--
			1	21 Dec (12:16)	H F	Grand Anse	0
250796	88	42	1	2 Dec (08:21)		Grand Anse	--
			1	17 Dec (07:59)	H	Ile aux Récifs**	19.13
			1	31 Dec (09:58)	H	Ile aux Récifs**	19.13
			1	13 Jan (06:35)	H F	Ile aux Récifs**	19.13
250797	90	27	1	2 Dec (14:34)		Grand Anse	--

Turtle ID	CCL (cm)	Days spent at breeding grounds post tag deployment (d)	Nests recorded (*n*)	Nest date (h)	Evidence	Nesting beach	Straight-line distance between first and subsequent nests (km)
			1	16 Dec (17:52)	H F P	Grand Anse	0.09
			1	28 Dec (16:28)	H F	Grand Anse	0

Table S4 Hawksbill turtles (*Eretmochelys imbricata*) that laid > 1 clutch were more likely to remigrate compared to those that laid 1 clutch in a previous season on Grand Anse, Frégate Island, Seychelles.

**Nesting season**	**Total individuals (*n*)**	**Resighted**	**Number of individuals (*n*)**	
			**1 clutch**	**> 1 clutch**
2017-18	25	Yes	12	5
		No	7	1
2018-19	20	Yes	7	5
		No	8	0

Table S5 Estimates of number of adult females using Grand Anse across varying proportions of clutches laid on alternative beaches under three clutch frequency values: (a) foot patrol-derived mean estimate across seasons (1.9 clutches), (b) foot patrol-derived maximum annual estimate (2.5 clutches), and (c) satellite telemetry-derived estimate (3.7 clutches)

Clutch frequency (n)	Proportion of clutches laid elsewhere	Estimated adult female abundance (n)
1.9	0	260
	0.1	289
	0.2	325
	0.3	372
	0.4	434
	0.5	521
	0.6	651
	0.7	868
	0.8	1302
	0.9	2605
2.5	0	198
	0.1	220
	0.2	247
	0.3	282
	0.4	330
	0.5	396
	0.6	495
	0.7	660
	0.8	990
	0.9	1980
3.7	0	134
	0.1	148
	0.2	167
	0.3	191
	0.4	222
	0.5	267
	0.6	334
	0.7	445
	0.8	668
	0.9	1337

Table S6 Global reports of five study-beach unfaithful sea turtle species (all except flatback turtles *Natator depressus* and Kemp's ridley turtles *Lepidochelys kempii*) with fidelity encompassing more than one nesting beach within or between seasons. Asterisk indicates that the exact alternative nesting location was not provided. Two asterisks indicate anecdotal observations in which the total number of turtles observed was equal to the number showing study-beach unfaithful behaviour. ST: Satellite telemetry, FT: Flipper tagging, GA: Genetic analysis, PID: Photo-identification, NA: Not available. Mainland* indicates mainland and barrier islands.

**Species**	**Region**	**Method**	**Primary nesting site**	**Survey length (km)**	**Geography type**	**Alternate sites (s)**	**Incidence of study-beach unfaithful individuals (%)**	**Total observed turtles (n)**	**Intra- or inter-season**	**Maximum distance between nests (km)**	**Reference**
Eretmochelys imbricata	Indo-Pacific	*ST*	*Frégate, Seychelles*	0.3	Continental islands	*Marianne and Ile aux Récifs, Seychelles*	*50.00*	*10*	*Intra-*	*27.3*	*This study*
		FT	Cousin, Seychelles	1.7	Continental islands	Praslin and Grand Soeur, Seychelles	1.45	275	Intra- and Inter-	2-27	Diamond, 1976
			Cousin, Seychelles	1.7	Continental islands	Cousine, Mahe, Praslin, Aride, and Curieuse, Seychelles	8.65	696	Intra-	2.8-13	Allen et al., 2010
			Milman Island, Australia	2.4	Continental islands	Sinclair, Douglas, Crocodile, Boydong Islands, Australia	1.67	2027	Intra- and Inter-	36	Limpus et al., 2008
		ST	Aukane Island, Australia	NA	Continental islands	Mimi, Layoak, Arden and Aureed Islands, Australia	22.22	9	Intra-	13	Barr et al., 2021
			Kerehikapa, Solomon Islands	NA	Oceanic islands	Sikopo, Solomon Islands	16.67	30	Intra-	8.5	Hamilton et al., 2021
	Atlantic	ST	St. Eustatius, Dutch Caribbean	1.6	Oceanic islands	Scrub Island, Anguilla and St Croix, US Virgin Islands (USVI)	50.00	2	Intra-	187	Esteban et al., 2015

Species	Region	Method	Primary nesting site	Survey length (km)	Geography type	Alternate sites (s)	Incidence of study-beach unfaithful individuals (%)	Total observed turtles (n)	Intra- or inter-season	Maximum distance between nests (km)	Reference
			Buck Island Reef National Monument (BIRNM), USVI	NA	Oceanic islands	St Croix, USVI	3.23	31	Intra-	~20	Sartain Iverson et al., 2016
		GA	Jumby Bay, Antigua	1	Oceanic islands	Mainland Antigua and BIRNM, USVI	**	4	Inter-	300	Levasseur et al., 2019
			Jumby Bay, Antigua	1	Oceanic islands	Other Antiguan beaches	0.39	256	Intra-	15	Levasseur et al., 2021
Chelonia mydas	Indo-Pacific	FT	Eastern Australia: North Great Barrier Reef (nGBR) and South Great Barrier Reef (sGBR)	NA	Continental islands	Within Eastern Australia	NA	NA	Intra-<br>Inter-	12-50 (nGBR)<br>17-188 (sGBR)<br>12-281 (nGBR)<br>17-266 (sGBR)	Dethmers et al., 2006
		ST	Raine Island, Australia	NA	Continental islands	Moulter Cay, Australia	5.13	39	Intra-	15	Hamann et al., 202 ; Perez et al., 2024
		FT/PID	Perhentian Islands, Malaysia	NA	Continental islands	Redang and other Perhentian beaches, Malaysia	10.53	57	Inter-	30	Long and Azmi, 2017
		ST	Diego Garcia index beach	2.3	Oceanic islands	Neighbouring beaches	5.56	18	Intra-	Within 10s of km	Esteban et al., 201
		FT	Tromelin	NA	Oceanic islands	Reunion	0.02	3766	Inter-	2250	Le Gall & Hughes, 1987
	Mediter-ranean Sea	ST	Alagadi beach, Cyprus	2	Mainland	Neighbouring beaches	10.34	29	Intra-	*	Snape et al., 2018

Species	Region	Method	Primary nesting site	Survey length (km)	Geography type	Alternate sites (s)	Incidence of study-beach unfaithful individuals (%)	Total observed turtles (n)	Intra- or inter-season	Maximum distance between nests (km)	Reference
	Atlantic	FT	Meio Island, Guinea-Bissau	NA	Continental islands	Poilao Island, Guinea-Bissau	19.06	419	Intra-	~13	Raposo et al., 2024
			Multiple beaches, Ascension Island	12.7	Oceanic islands	Multiple beaches, Ascension Island	15.10	371	Intra- and Inter-	1-4	Mortimer & Portier 1989
		ST	Long Beach, Ascension Island	NA	Oceanic islands	Deadman's Beach, English Bay, Payne Point, Pan Am Beach, Ascension Island	5.00	40	Intra-	1-4	Weber et al., 2013
		FT	Atol das Rocas, Brazil	1.5	Oceanic islands	Fernando de Noronha, Brazil	0.54	924	Inter-	~150	Bellini et al., 2012
			Bigisanti, Suriname	NA	Mainland	Galibi, Suriname	2.11	1423	Intra-	85	Schulz, 1971
			Playa Carite, Mona Island, Puerto Rico	NA	Oceanic islands	Aves Island, Venezuela	**	1	Intra-	560	Kontos et al., 1988
		ST	Tortuguero, Costa Rica	4	Mainland	Nicaragua	10.00	10	Intra-	135	Troëng et al., 2005
			St. Eustatius, Dutch Caribbean	1.6	Oceanic islands	Zeelandia beach, St. Eustatius, Dutch Caribbean	66.67	3	Intra-	1	Esteban et al., 2015
			St. Eustatius, Dutch Caribbean	1.6		St. Kitts, Dutch Caribbean	**	1	Intra-	21.8	
		FT	Tortuguero, Costa Rica	4	Mainland	Chiriquí, Panama	**	1	NA	260	Esteban et al., 2015
Dermochelys coriacea	Indo-Pacific	ST	Kamiali, Papua New Guinea	2	Continental islands	Morobe coast and Huan Peninsula, Papua New Guinea	21.05	19	Intra-	~100	Benson et al., 2007

Species	Region	Method	Primary nesting site	Survey length (km)	Geography type	Alternate sites (s)	Incidence of study-beach unfaithful individuals (%)	Total observed turtles (n)	Intra- or inter-season	Maximum distance between nests (km)	Reference
	Atlantic	FT	Juno Beach, Florida, US	19.4	Mainland	Other Florida and Georgia beaches, US	7.08	466	Intra-	463.5	Stewart et al., 2014
		ST	French Guiana, Grenada, Gabon,	NA	Mainland	Other French Guiana and Suriname beaches	53.13	32	Intra-	19.8	Georges et al., 200
		FT	Between countries in Wider Caribbean Region	NA	Mainland / Oceanic islands	Between countries in Wider Caribbean Region	2.82	3151	Intra-<br>Inter-	369 (consecutive nests)<br>532 (non-consecutive nests)<br>1849 km	Horrocks et al., 2016
			Sandy Point, St. Croix, US Virgin Islands	6.2	Oceanic islands	Manchenil Bay Beach, St. Croix, US Virgin Islands and Culebra, Puerto Rico	4.47	132	Intra-	20-85	Eckert et al., 1989
			Bigisanti, Suriname	NA	Mainland	French Guiana	8.54	82	Intra-	120	Schulz, 1971
Caretta caretta	Indo-Pacific	FT	Nagasaki, Japan	15.9	Continental islands	Miyazaki, Japan	3.70	27	Intra-	377	Iwamoto et al., 1985
	Mediterranean Sea	ST	Alagadi beach, Cyprus	2	Mainland	Neighbouring beaches	16.67	36	Intra-	20	Snape et al., 2018
			Potamakia beach, Cephalonia, Greece	2.7	Continental islands	Other Cephalonia beaches, Greece	100.00	1	Intra-	8.7	Hays et al., 1991
		FT	Potamakia beach, Cephalonia, Greece	2.7	Continental islands	Other Cephalonia and Zakynthos beaches, Greece	56.50	23	Intra-	~50	Hays & Sutherland, 1991

Species	Region	Method	Primary nesting site	Survey length (km)	Geography type	Alternate sites (s)	Incidence of study-beach unfaithful individuals (%)	Total observed turtles (n)	Intra- or inter-season	Maximum distance between nests (km)	Reference
			Cephalonia, Greece	NA	Continental islands	Peloponesse, Greece	NA	NA	NA	~60-200	Margaritoulis, 1998
	Atlantic	FT	Cape Verde	15	Oceanic islands	Neighbouring beaches	NA	NA	Intra-	130	Marco et al., 2011
			João Barrosa beach, Boa Vista, Cape Verde	NA	Oceanic islands	Ervatão and Calheta Lajedo Teixeira beaches, Boa Vista, Cape Verde	17.97	1296	Inter-	15	Martins et al., 2022
			West Florida, US	NA	Mainland	East Florida, US	**	2	Intra- and Inter-	550	LeBuff, 1974
			Hutchinson Island, Florida, US	11.25	Mainland*	Jupiter Island, Melbourne Beach, Florida, US	4.62	260	Intra-	4.6	Worth & Smith, 1976
			Little Cumberland Island, Georgia, US	NA	Mainland*	Jekyll and Cumberland Islands, Georgia, US	2.00	647	Inter	> 50	Bell & Richardson, 1978
		FT	Kiawah Island, South Carolina, US	16	Mainland*	Fripp and South Islands, South Carolina, US	0.95	211	NA	3.2 ± 1.8 (mean ± SD)	Talbert et al., 1980
			North Carolina, US	NA	Mainland	Florida, US	**	1	Intra-	725	Stoneburner & Ehrhart, 1981
			Hutchinson Island, Florida, US	11.25	Mainland*	Neighbouring beaches	NA	NA	Intra-	15.9	Williams-Walls et al., 1983
		FT	Melbourne beach, Florida, US	11.2	Mainland	Other Florida beaches, US	2.82	2910	Intra-<br>Inter-	290<br>700	Bjorndal et al., 1983

Species	Region	Method	Primary nesting site	Survey length (km)	Geography type	Alternate sites (s)	Incidence of study-beach unfaithful individuals (%)	Total observed turtles (n)	Intra- or inter-season	Maximum distance between nests (km)	Reference
			Bald Head Island, North Carolina, US	15.3	Mainland*	Beaches between North Carolina and Florida, US	2.31	477	Inter-	~1-800	Hawkes et al., 2005
			Cape San Blas, Florida, US	5	Mainland	Gulf Islands National Seashore, Florida, US	0.90	111	Intra-	250	Lamont & Carthy, 2007
			Wassaw National Wildlife Refuge (NWR), Georgia, US	10.7	Mainland*	Between Onslow beach, NC & Cape Canaveral, FL, US. Predominantly Blackbeard and Ossabaw Islands, Georgia	9.18	1295	Inter-	400	Williams & Frick, 2008
		ST	Casey Key, Florida, US	6	Mainland	Other Florida beaches, US	45.96	285	Intra	109.1	Tucker, 2010
			Bon Secour & Baldwin County, Alabama & St. Joseph's Peninsula, Florida, US	35	Mainland	Beaches between Alabama & Florida	46.15	39	Intra-	402	Hart et al., 2013
		FT/GA	Between Maryland & Georgia, US	>1000	Mainland*	Between Maryland & Georgia, US	54.00	NA	Intra-	723.8	Shamblin et al., 2017
		FT/GA	Wassaw NWR, Georgia, US	>1000	Mainland*	Between Maryland & Georgia, US	NA	NA	NA	Up to 800	Pfaller et al., 2022
		ST	Busca Vida, Brazil	215	Mainland	Praia do Forte, Brazil	10.00	10	Intra-	41	Marcovaldi et al., 2010
Lepidochelys olivacea	Indo-Pacific	FT	Gahirmatha, India	6	Mainland	Devi and Rushikulya, India	0.93	1070	Inter-	320	Tripathy & Pandav, 2007
			Bigisanti, Suriname	NA	Mainland	Galibi, Suriname	0.43	690	Intra-	85	Schulz, 1971

Species	Region	Method	Primary nesting site	Survey length (km)	Geography type	Alternate sites (s)	Incidence of study-beach unfaithful individuals (%)	Total observed turtles (n)	Intra- or inter-season	Maximum distance between nests (km)	Reference
	Atlantic		Corozalito, Costa Rica	11.4	Mainland	Bejuco, San Miguel and Costa de Oro beaches	17.8	555	Intra-<br>Inter-	15	Fusté et al., 2026

Table S7 One-time nesting turtles, i.e. individuals recorded nesting only once on a particular beach, were identified through saturated tagging efforts across sites.

Species	Monitored beach	One-time nesting individuals (%)	Reference
Dermochelys coriacea	Culebra, Puerto Rico	8.8	Tucker & Frazer, 1991
	Juno beach, Florida, US	43	Stewart et al., 2014
Caretta caretta	Kiawah Island, US	26.3-60	Talbert et al., 1980
	Potomakia beach, Cephalonia, Greece	56.5	Hays & Sutherland, 1991
	Bald Head Island, US	55.9	Webster & Cook, 2001
	Wassaw NWR, Georgia, US	~60	Williams & Frick, 2008

References

Allen, Z. C., Shah, N. J., Grant, A., Derand, G., & Bell, D. (2010). Hawksbill turtle monitoring in Cousin Island Special Reserve, Seychelles: an eight-fold increase in annual nesting numbers. *Endangered Species Research, 11*, 195–200. https://doi.org/10.3354/esr00281

Barr, C. E., Hamann, M., Shimada, T., Bell, I., Limpus, C. J., & Ferguson, J. (2021). Post-nesting movements and feeding ground distribution by the hawksbill turtle (*Eretmochelys imbricata*) from rookeries in the Torres Strait. *Wildlife Research, 48*, 598–608. https://doi.org/10.1071/WR20183

Bell, R., & Richardson, J. I. (1978). An analysis of tag recoveries from loggerhead sea turtles (*Caretta caretta*) nesting on Little Cumberland Island, Georgia. *Florida Marine Research Publications, 33*, 20–24. Downloaded from: http://www.seaturtle.org/PDF/HendersonGE_1978_FMRTechReport.pdf#page=27

Bellini, C., Santos, A. J. B., Grossman, A., Marcovaldi, M. A., & Barata, P. C. R. (2012). Green turtle (*Chelonia mydas*) nesting on Atol das Rocas, north-eastern Brazil, 1990-2008. *Journal of Marine Biological Association of the United Kingdom, 93*, 1117–1132. https://doi.org/10.1017/S002531541200046X

Benson, S. R., Kisokau, K. M., Ambio, L., Rei, V., Dutton, P. H., & Parker, D. (2007). Beach use, internesting movement, and migration of leatherback turtles, *Dermochelys coriacea*, nesting on the north coast of Papua New Guinea. *Chelonian Conservation and Biology, 6*, 7–14. https://doi.org/10.2744/1071-8443(2007)6[7:BUIMAM]2.0.CO;2

Bjorndal, K. A., Meylan, A. B., & Turner, B. J. (1983). Sea turtles nesting at Melbourne Beach, Florida, I. Size, growth and reproductive biology. *Biological Conservation, 26*, 65–77. https://doi.org/10.1016/0006-3207(83)90049-6

Dethmers, K. E. M., Broderick, D., Moritz, C., Fitzsimmons, N. N., Limpus, C. J., Lavery, S., Whiting, S., Guinea, M., Prince, R. I. T., & Kennett, R. (2006). The genetic structure of Australasian green turtles (*Chelonia mydas*): exploring the geographical scale of genetic

exchange. *Molecular Ecology, 15*, 3931–3946. https://doi.org/10.1111/j.1365-294X.2006.03070.x

Diamond, A. W. (1976). Breeding biology and conservation of hawksbill turtles, *Eretmochelys imbricata* L., on Cousin island, Seychelles. *Biological Conservation, 9*, 199–215. https://doi.org/10.1016/0006-3207(76)90010-0

Eckert, K. L., Eckert, S. A., Adams, T. W., & Tucker, A. D. (1989). Inter-nesting migrations by leatherback sea turtles (*Dermochelys coriacea*) in the West Indies. *Herpetologica, 45*, 190–194. https://www.jstor.org/stable/3892161

Esteban, N., van Dam, R. P., Harrison, E., Herrera, A., & Berkel, J. (2015). Green and hawksbill turtles in the Lesser Antilles demonstrate behavioural plasticity in inter-nesting behaviour and post-nesting migration. *Marine Biology, 162,* 1153–1163. https://doi.org/10.1007/s00227-015-2656-2

Esteban, N., Mortimer, J. A., & Hays, G. C. (2017). How number of nesting sea turtles can be overestimated by nearly a factor of two. *Proceedings of the Royal Society B, 284*, 20162581. http://dx.doi.org/10.1098/rspb.2016.2581

Fusté, R., Mejías-Balsalobre, C., Webster, E. G., Naranjo, I., Arauz, R., & Rojas-Cañizales, D. (2026). Inter-beach movements and inter-nesting periods of olive ridley sea turtles relate to nesting behavior in a nascent arribada beach. *Marine Biology, 173,* 117. https://doi.org/10.1007/s00227-026-04856-z

Georges, J.-Y., Fossette, S., Billes, A., Ferraroli, S., Fretey, J., Grémillet, D., Le Maho, Y., Myers, A. E., Tanaka, H., & Hays, G. C. (2007). Meta-analysis of movements in Atlantic leatherback turtles during the nesting season: conservation implications. *Marine Ecological Progress Series, 338*, 225–232. https://doi.org/10.3354/meps338225

Hamann, M., Shimada, T., Duce, S., Foster, A., Tsz Ying To, A., & Limpus, C. (2022). Patterns of nesting behaviour and nesting success for green turtles at Raine Island, Australia. *Endangered Species Research, 47*, 217–229. https://doi.org/10.3354/esr01175

Hamilton, R. J., Desbiens, A., Pita, J., Brown, C. J., Vuto, S., Atu, W., James, R., Waldie, P., & Limpus, C. (2021). Satellite tracking improves conservation outcomes for nesting hawksbill turtles in Solomon Islands. *Biological Conservation, 261,* 109240. https://doi.org/10.1016/j.biocon.2021.109240

Hart, K. M., Lamont, M. M., Sartain, A. R., Fujisaki, I., & Stephens, B. S. (2013). Movements and habitat-use of loggerhead sea turtles in the Northern Gulf of Mexico during the reproductive period. *PLoS ONE, 8,* e66921. https://doi.org/10.1371/journal.pone.0066921

Hawkes, L. A., Broderick, A. C., Godfrey, M. H., & Godley, B. J. (2005). Status of nesting loggerhead turtles, *Caretta caretta*, at Bald Head Island (North Carolina, USA) after 24 years of intensive monitoring and conservation. *Oryx, 39,* 65–72. https://doi.org/10.1017/S0030605305000116

Hays, G. C., & Sutherland, J. M. (1991). Remigration and beach fidelity of loggerhead turtles nesting on the Island of Cephalonia, Greece. *Journal of Herpetology, 25*, 232–233. https://www.jstor.org/stable/1564660

Hays, G. C., Webb, P. I., Hayes, J. P., Priede, I. G., & French, J. (1991). Satellite tracking of a loggerhead turtle (*Caretta caretta*) in the Mediterranean. *Journal of Marine Biological Association of the United Kingdom, 71*, 743–746. https://doi.org/10.1017/S0025315400053261

Horrocks, J. A., Stapleton, S., Guada, H., Lloyd, C., Harris, E., Fastigi, M., Berkel, J., Stewart, K., Gumbs, J., & Eckert, K. L. (2016). International movements of adult female leatherback turtles in the Caribbean: results from tag recovery data (2002-2013). *Endangered Species Research*, 29, 279–287. https://doi.org/10.3354/esr00718

Iwamoto, T., Ishii, M., Nakashima, Y., Takeshita, H., & Itoh, A. (1985). Nesting cycles and migrations of the loggerhead sea turtle in Miyazaki, Japan. *Japanese Journal of Ecology, 35*, 505–511. https://doi.org/10.18960/seitai.35.4_505

Kontos, A., Eckert, S., Eckert, K., Gomez, J., Lee, R., & Van Dam, R. (1988). Inter-island migration of nesting green turtle, *Chelonia mydas*. *Marine Turtle Newsletter, 42*, 10-11.

Lamont, M. M., & Carthy, R. R. (2007). Response of nesting sea turtles to Barrier Island dynamics. *Chelonian Conservation & Biology, 6*, 206–212. https://doi.org/10.2744/1071-8443(2007)6[206:RONSTT]2.0.CO;2

Levasseur, K. E., Stapleton, S. P., Fuller, M. C., & Quattro, J. M. (2019). Exceptionally high natal homing precision in hawksbill sea turtles to insular rookeries of the Caribbean. *Marine Ecological Progress Series, 620*, 155–171. https://doi.org/10.3354/meps12957

Levasseur, K. E., Stapleton, S. P., & Quattro, J. M. (2021). Precise natal homing and an estimate of age at sexual maturity in hawksbill turtles. *Animal Conservation, 24,* 523–535. https://doi.org/10.1111/acv.12657

LeBuff Jr., C. R. (1974). Unusual nesting relocation in the loggerhead turtle, *Caretta caretta*. *Herpetologica, 30,* 29–31.

Le Gall, J.-Y., & Hughes, G. R. (1987). Migrations de la tortue verte *Chelonia mydas* dans l'Ocean Indien Sud-Ouest observees a partir des marquages sur les de ponte Europa et Tromelin (1970-1985). *Amphibia-Reptilia, 8*, 277–282. https://doi.org/10.1163/156853887X00315

Limpus, C. J., Miller, J. D., Guinea, M., & Whiting, S. (2008). Australian hawksbill turtle population dynamics project. Queensland: Environmental Protection Agency. Pp. 140.

Long, S.-L., & Azmi, N. A. (2017). Using photographic identification to monitor sea turtle populations at Perhentian Islands Marine Park in Malaysia. *Herpetological Conservation & Biology, 12*, 350–366.

Marco, A., Pérez, E. A., Argüello, C. M., Martins, S., Araujo, S., & Jurado, L. F. L. (2011). The international importance of the archipelago of Cape Verde for marine turtles, in particular

the loggerhead turtle *Caretta caretta*. *Zoologia Caboverdiana, 2*, 1–11. http://hdl.handle.net/10553/18267

Marcovaldi, M. A., Lopez, G. G., Soares, L. S., Lima, E. H. S. M., Thomé, J. C. A., & Almeida, A. P. (2010). Satellite-tracking of female loggerhead turtles highlights fidelity behaviour in northeastern Brazil. *Endangered Species Research, 12*, 263–272. https://doi.org/10.3354/esr00308

Margaritoulis, D. (1998). Interchange of nesting loggerheads among Greek beaches. In Proceedings of the Seventeenth Annual Sea Turtle Symposium, edited by S. P. Epperly and J. Braun. NOAA Technical Memorandum NMPS-SEFC-415, Orlando.

Martins, S., Cardona, L., Abella, E., Silva, E., Loureiro, N. S., Roast, M., & Marco, A. (2022). Effect of body size on the long-term reproductive output of eastern Atlantic loggerhead turtles *Caretta caretta*. *Endangered Species Research, 48*, 175–189. https://doi.org/10.3354/esr01198

Mortimer, J. A., & Portier, K. M. (1989). Reproductive homing and interesting behavior of the green turtle (*Chelonia mydas*) at Ascension Island, South Atlantic Ocean. *Copeia, 1989*, 962–977. https://doi.org/10.2307/1445982

Perez, M., Limpus, C., Shimada, T., McDonald, S., Coffee, O., Hinchliffe, E., & Hamann, M. (2024). Inter-nesting habitat use by green turtles *Chelonia mydas* in the Great Barrier Reef. *Endangered Species Research, 54*, 225–238. https://doi.org/10.3354/esr01340

Pfaller, J. B., Weaver, S. M., Williams, K. L., Dodd, M. G., Godfrey, M. H., Griffin, D. B., Pate, S. M., Glen, C. G., Nairn, C. J., & Shamblin, B. M. (2022). One beach amongst many: how weak fidelity to a focal nesting site can bias demographic rates in marine turtles. *Marine Biology, 169,* 8. https://doi.org/10.1007/s00227-021-03991-z

Raposo, C., Rebelo, R., Catry, P., Ferreira-Airaud, M. B., Barbosa, C., Garcia, T. B., Regalla, A., Sampaio, M. S., & Patrício, A. R. (2024). Inter-island nesting dynamics and clutch survival of green turtles *Chelonia mydas* within a marine protected area in the Bijagós Archipelago, West Africa. *Marine Biology, 171*, 143. https://doi.org/10.1007/s00227-024-04463-w

Sartain Iverson, A. R., Hart, K. M., Fujisaki, I., Cherkiss, M. S., Pollock, C., Lundgren, I., & Hillis-Starr, Z. (2016). Hawksbill satellite-tracking case study: Implications for remigration interval and population estimates. *Marine Turtle Newsletter, 148*, 2–7.

Schulz, J. P. (1971). Situation report on marine turtles nesting in Surinam. In Marine Turtles. IUCN Publications New Series, Supplementary Paper 31, 68–74. International Union for Conservation of Nature and Natural Resources, Morges, Switzerland.

Shamblin, B. M., Dodd, M. G., Griffin, D. B., Pate, S. M., Godfrey, M. H., Coyne, M. S., Williams, K. L., Pfaller, J. B., Ondich, B. L., Andrews, K. M., Boettcher, R., & Nairn, C. J. (2017). Improved female abundance and reproductive parameter estimates through subpopulation-scale genetic capture-recapture of loggerhead turtles. *Marine Biology, 164*, 138. https://doi.org/10.1007/s00227-017-3166-1

Snape, R. T. E., Bradshaw, P. J., Broderick, A. C., Fuller, W. J., Stokes, K. L., & Godley, B. J. (2018). Off-the-shelf GPS technology to inform marine protected areas for marine turtles. *Biological Conservation, 227,* 301–309. https://doi.org/10.1016/j.biocon.2018.09.029

Stewart, K. R., Martin, K. J., Johnson, C., Desjardin, N., Eckert, S. A., & Crowder, L. B. (2014). Increased nesting, good survival and variable site fidelity for leatherback turtles in Florida, USA. *Biological Conservation, 176,* 117–125. http://dx.doi.org/10.1016/j.biocon.2014.05.008

Stoneburner, D. L., & Ehrhart, L. M. (1981). Observations on *Caretta c. caretta*: a record internesting migration in the Atlantic. *Herpetological Reviews, 12*, 66.

Talbert, O. R. Jr., Stancky, S. E., Dean, J. M., & Will, J. M. (1980). Nesting activity of the loggerhead turtle (*Caretta caretta*) in South Carolina I: a rookery in transition. *Copeia, 1980*, 709–718. https://www.jstor.org/stable/1444448

Tripathy, B., & Pandav, B. (2007). Beach fidelity and internesting movements of olive ridley turtles (*Lepidochelys olivacea*) at Rushikulya, India. *Herpetological Conservation & Biology, 3*, 40–45. Downloaded from: http://www.seaturtle.org/PDF/TripathyB_2008_HerpConservBiol.pdf

Troëng, S., Evans, D. R., Harrison, E., & Lageaux, C. J. (2005). Migration of green turtles from Tortuguero, Costa Rica. *Marine Biology, 148,* 435–447. https://doi.org/10.1007/s00227-005-0076-4

Tucker, A. D. (2010). Nest site fidelity and clutch frequency of loggerhead turtles are better elucidated by satellite telemetry than by nocturnal tagging efforts: implications for stock estimation. *Journal of Experimental Marine Biology & Ecology, 383*, 48–55. https://doi.org/10.1016/j.jembe.2009.11.009

Tucker, A. D., & Frazer, N. B. (1991). Reproductive variation in leatherback turtles, *Dermochelys coriacea*, at Culebra National Wildlife Refuge, Puerto Rico. *Herpetologica, 47*, 115–125. https://www.jstor.org/stable/3892822

Weber, N., Weber, S. B., Godley, B. J., Ellick, J., Witt, M., & Broderick, A. C. (2013). Telemetry as a tool for improving estimates of marine turtle abundance. *Biological Conservation, 167*, 90–96. https://dx.doi.org/10.1016/j.biocon.2013.07.030

Webster, W. D., & Cook, K. A. (2001). Intraseasonal nesting activity of loggerhead sea turtles (*Caretta caretta*) in Southeastern North Carolina. *American Midland Naturalist, 145*, 66–73. https://doi.org/stable/3083080

Williams, K. L., & Frick, M. G. (2008). Tag returns from loggerhead turtles from Wassaw Island, GA. *Southeast Naturalist, 7*, 165–172. www.jstor.com/stable/20203982

Williams-Walls, N., O'Hara, J., Gallagher, R. M., Worth, D. F., Peery, B. D., & Wilcox, J. R. (1983). Spatial and temporal trends of sea turtle nesting on Hutchinson Island, Florida, 1971-1979. *Bulletin of Marine Science, 33*, 55–66.

Worth, D. F., & Smith, J. B. (1976). Marine turtle nesting on Hutchinson Island, Florida, in 1973. *Florida Marine Research Publications, 18*, 1–14
